# Phosphoproteomic Analysis of Platelets Activated by Pro-Thrombotic Oxidized Phospholipids and Thrombin

**DOI:** 10.1371/journal.pone.0084488

**Published:** 2014-01-06

**Authors:** Alejandro Zimman, Bjoern Titz, Evangelia Komisopoulou, Sudipta Biswas, Thomas G. Graeber, Eugene A. Podrez

**Affiliations:** 1 Department of Molecular Cardiology, Lerner Research Institute, Cleveland Clinic, Cleveland, Ohio, United States of America; 2 Crump Institute for Molecular Imaging, Department of Molecular and Medical Pharmacology, Institute for Molecular Medicine, Jonsson Comprehensive Cancer Center and California NanoSystems Institute, David Geffen School of Medicine, University of California Los Angeles, Los Angeles, California, United States of America; Universität Regensburg, Germany

## Abstract

Specific oxidized phospholipids (oxPC_CD36_) promote platelet hyper-reactivity and thrombosis in hyperlipidemia via the scavenger receptor CD36, however the signaling pathway(s) induced in platelets by oxPC_CD36_ are not well defined. We have employed mass spectrometry-based tyrosine, serine, and threonine phosphoproteomics for the unbiased analysis of platelet signaling pathways induced by oxPC_CD36_ as well as by the strong physiological agonist thrombin. oxPC_CD36_ and thrombin induced differential phosphorylation of 115 proteins (162 phosphorylation sites) and 181 proteins (334 phosphorylation sites) respectively. Most of the phosphoproteome changes induced by either agonist have never been reported in platelets; thus they provide candidates in the study of platelet signaling. Bioinformatic analyses of protein phosphorylation dependent responses were used to categorize preferential motifs for (de)phosphorylation, predict pathways and kinase activity, and construct a phosphoproteome network regulating integrin activation. A putative signaling pathway involving Src-family kinases, SYK, and PLCγ2 was identified in platelets activated by oxPC_CD36_. Subsequent *ex vivo* studies in human platelets demonstrated that this pathway is downstream of the scavenger receptor CD36 and is critical for platelet activation by oxPC_CD36_. Our results provide multiple insights into the mechanism of platelet activation and specifically in platelet regulation by oxPC_CD36_.

## Introduction

Dyslipidemia is associated with oxidative stress and platelet hyper-reactivity, a condition that increases the risk of thrombotic complications in cardiovascular pathologies [1], [2]. Evidence suggests that oxidative stress in dyslipidemia promotes accumulation of specific oxidized phospholipids in circulation, leading to increased platelet activation responses and contributing to a prothrombotic state. Biologically active oxidized phospholipids are present in oxidized lipoproteins, apoptotic cells, atherosclerotic lesions, and they accumulate in significant amounts in circulation [3]–[6]. Oxidized choline glycerophospholipids are markedly increased in plasma of hyperlipidemic mice and in plasma of subjects with low HDL level, and promote platelet activation and hyper-reactivity [3]. Selective removal of oxidized phospholipids from plasma prevents platelet reactivity [7], providing further evidence for their contribution to platelet hyper-reactivity in dyslipidemia.

We previously showed that the scavenger receptor CD36 is the major receptor on platelets that promotes platelet reactivity in hyperlipidemic conditions in response to oxidized phospholipids [3]. *In vitro*, CD36 mediates platelet activation by a group of specific oxidized phospholipids (oxPC_CD36_) derived from the oxidation of 1-palmitoyl-2-arachidonyl-phosphatidylcholine (PAPC) and 1-palmitoyl-2-linoleoyl-phosphatidylcholine (PLPC). Electrostatic interaction of the negatively charged carboxylate at sn-2 position of oxPC_CD36_ with lysine [8], [9] and covalent binding of the electrophilic phospholipid to cysteine, lysine, or histidine residues [10], [11] serve as the basis for the recognition of oxidized phospholipids by CD36 and other proteins. Although the receptor responsible for platelet activation by oxidized phospholipids and the binding mechanism are established, very little is known regarding the signaling pathways downstream of CD36 in platelets. Some progress has been made in the signaling mechanisms initiated by oxidized LDL (oxLDL) in platelets [12], [13]. However, oxLDL contains a complex mixture of agonists (i.e. oxPC_CD36_, amyloid-like structures, lysophosphatidylcholine, oxysterols, PAF receptor activating-oxidized lipids) that can lead to the activation of several independent signaling mechanisms [14]–[17]. Given the important role of oxidized phospholipids in platelet hyper-reactivity, it is essential to establish the specific signaling mechanism(s) that control platelet activation by these ligands.

Phosphoproteomic studies are particularly useful in recognizing platelet signaling mechanisms, since genomic approaches are limited in anucleated cells. Currently, phosphoproteomics based on mass spectrometry provide the most comprehensive analysis for identification and quantitation of phosphorylation sites from diverse biological samples [18]–[20]. We have previously employed a phosphoproteomic protocol based on the digestion of protein lysate, enrichment of phosphopeptides in solution, and identification of peptides by liquid chromatography-tandem mass spectrometry to study changes in protein phosphorylation induced by a complex mixture of oxidized products of PAPC in aortic endothelial cells [21]. Results from this study showed phosphorylation-dependent responses of over 220 proteins and provided new evidence for the participation of the receptor tyrosine kinase VEGFR2 in the regulation of pro-inflammatory and coagulation genes by oxidized phospholipids in endothelial cells [22]. In the current study, we compared changes in phosphorylation between platelets activated by oxidized phospholipids to platelets activated by the strong agonist thrombin, in order to establish common and differential signaling mechanisms. Thus, our study on phosphorylation-dependent responses in platelets adds new information to protein function in platelet homeostasis and disease. Our phosphoproteomic results led us to construct the phosphoproteome network regulating integrin activation, and to determine that the pathway Src-family kinase, Spleen tyrosine kinase, and phospholipase PLCγ2 is responsible for platelet activation by oxidized phospholipids.

## Materials and Methods

### Materials

Prostaglandin I_2_ (PGI_2_) sodium salt, Sepharose 2B, BSA, DMSO, and U73122 were purchased from Sigma-Aldrich (St Louis, MO). Human α-thrombin was from Enzyme Research Laboratories (South Bend, IN) and ADP from Chrono-log (Havertown, PA). PE-conjugated anti–P-selectin antibody was obtained from Becton Dickinson (San Jose, CA) and mouse anti-CD36 (clone FA6-152) was purchased from Beckman Coulter (Brea, CA). PP2, PP3, PD98059, and BAY61-3606 were obtained from EMD Millipore (Billerica, MA). PLPC (1-palmitoyl-2-linoleoyl-*sn*-glycero-3-phosphocholine) was from Avanti Polar Lipids (Alabaster, AL). KODA-PC (9-keto-12-oxo-10-dodecenoic acid ester of 2-lyso-phosphocholine) was synthesized as described previously [23]. PLPC and KODA-PC were resuspended in Tyrode's buffer (without BSA and glucose) to prepare a 500 µM stock solution just before the incubation with platelets as described previously [5].

### Ethics statement

All experiments done with human blood were approved by the Cleveland Clinic's Institutional Review Board. Written informed consent was obtained in accordance with the Declaration of Helsinki.

### Platelet isolation

Human venous blood was drawn from healthy donors into acid-citrate-dextrose solution (ACD; 85 mM tri-sodium citrate, 65 mM citric acid, and 111 mM D-glucose; pH 4.6). PGI_2_ was added after blood collection into ACD to a final concentration 0.1 µg/mL. Platelet-rich plasma (PRP) was separated by centrifugation at 150 g (15 min, 22°C). Platelets were pelleted from PRP by centrifugation at 930 g (10 min, 22°C), resuspended in Tyrode's buffer (137 mM NaCl, 12 mM NaHCO_3_, 2.5 mM KCl, 10 mM HEPES, 0.1 % BSA, 0.1 % glucose, pH 7.4), and further purified by gel filtration using a Sepharose 2B column. The concentration of platelets was determined using Cellometer M10 (Nexcelom Bioscience, Lawrence, MA) and adjusted to concentrations indicated with Tyrode's buffer. CaCl_2_ and MgCl_2_ were added to a final concentration of 2 mM and 1 mM, respectively, for phosphoproteomic and flow cytometry experiments.

### Phosphoproteomic and bioinformatic analysis of human platelets

Human platelets isolated by gel filtration (2.7×10^8^/mL) were incubated in Tyrode's buffer with 50 µM KODA-PC or PLPC (as control) for 30 min at 37°C. Platelets were then centrifuged at 3,700 g (10 min, 35°C) and lysed in 8 M urea, 50 mM Tris-HCl (pH 7.4), 1 mM Na_3_VO_4_, and 1 mM NaF with sonication. Phosphoproteome changes induced by the agonist from a single biological experiment were assessed based on trypsin digestion of the protein lysate, phosphopeptide enrichment, mass spectrometry analysis, chromatography alignment, quantitation, and bioinformatics and enrichment analysis as described in Materials and Methods S1 and in our previous publications [21], [24]. The same protocol was applied when studying platelet activation by thrombin, where gel-filtered platelets were incubated in Tyrode's buffer with 0.05 U/mL thrombin or in buffer alone (Resting) for 3 min at 37°C. The mass spectrometry proteomics data have been deposited to the ProteomeXchange Consortium (http://proteomecentral.proteomexchange.org) via the PRIDE partner repository [25] with the dataset identifier PXD000451. Annotated spectra can be found in Annotated Spectra S1, Annotated Spectra S2, Annotated Spectra S3, Annotated Spectra S4, and Annotated Spectra S5.

### Immunoblotting

Platelets isolated by gel filtration (2×10^8^/mL) and treated with indicated inhibitors and agonists were pelleted at 960 g (5 min, 37°C). The platelets were then lysed for 30 min on ice with 50 mM Tris-HCl (pH 7.4), 1 % IGEPAL CA-630, 0.25 % sodium deoxycholate, 150 mM NaCl, 1 mM EDTA, 1 mM PMSF, 1 µg/mL aprotinin, 1 µg/mL leupeptin, 1 µg/mL pepstatin, 1 mM Na_3_VO_4_, and 1 mM NaF. Proteins were separated by SDS-PAGE, transferred to PVDF membranes, and probed with rabbit anti-human phospho-PLCγ2 Tyr759, phospho-PLCγ2 Tyr1217, phospho-SYK Tyr323, phospho-SYK Tyr525/526 (C87C1), or phospho-Src family Tyr416 (Cell Signaling, Danvers, MA). The membranes were stripped and then probed for normalization with rabbit anti-human SYK (C-20) (Santa Cruz Biotechnology, Santa Cruz, CA) or PLCγ2 (Cell Signaling).

### Flow cytometry analysis

Human platelets isolated by gel filtration (2×10^7^/mL) were incubated in Tyrode's buffer with the indicated inhibitors for 30 min and then with agonists for another 30 min. Platelets were incubated with PE-conjugated anti–P-selectin antibody and then fixed with 2 % formaldehyde in PBS. Data was acquired using a FACSCalibur instrument (Becton Dickinson, San Jose, CA) and analyzed using FlowJo (Tree Star, Ashland, OR). Data are presented as mean ±SD. Significant changes were determined using one-way ANOVA with Bonferroni post-test. Significance was accepted at the level of P<0.05.

## Results

### System-wide phosphoproteomic analysis of platelets activated by the oxidized phospholipid KODA-PC and thrombin reveals common and differential signaling events

**Figure 1:**
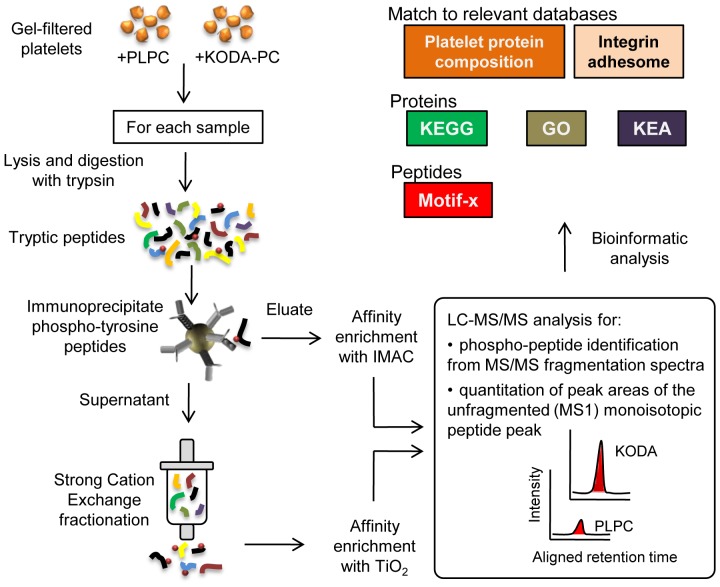
Phosphoproteomic analysis of platelets activated by the oxidized phospholipid KODA-PC and thrombin. Human platelets isolated by gel filtration were incubated with the oxidized phospholipid KODA-PC, or PLPC (as control). Platelets were then lysed and the proteins digested with trypsin. Phospho-tyrosine peptides were enriched from tryptic peptides with 4G10 antibody followed by metal affinity enrichment (IMAC). The supernatant leftover after immunoprecipitation was fractionated by Strong Cation Exchange (SCX) to separate phospho-serine/threonine peptides from unphosphorylated peptides. Fractions collected by SCX were further enriched for phosphopeptides using TiO_2_ metal affinity. Phosphopeptide-enriched samples were analyzed by LC-MS/MS for identification and quantitation. A detailed description of the method can be found in the Materials and Methods S1. Differences in protein phosphorylation between platelets incubated with KODA-PC and PLPC as determined by the phosphoproteomic method were used for analysis at the peptide level (Motif-X), protein level (GO-term, KEGG-pathway, and Kinase enrichment analysis-KEA), and compared to databases relevant to platelet biology. The same methodology was employed to study differences in protein phosphorylation between thrombin-induced and resting platelets.

oxPC_CD36_ represent a family of oxidized choline glycerophospholipids accumulating *in vivo* in dyslipidemia and oxidative stress that possess several proatherogenic properties, including platelet activation [3]. We investigated the signaling pathways induced by oxPC_CD36_ in platelets using KODA-PC as one of the most abundant and active representatives of the oxPC_CD36_ family [3]. Initial immunoblotting studies using pan-specific anti-phosphotyrosine and phospho-serine PKC substrate antibodies detected changes in protein phosphorylation induced by KODA-PC in isolated human platelets (Figure S1 in File S1). For further comprehensive investigation of protein phosphorylation, we employed a label-free phosphoproteomic approach based on the enrichment of relatively less abundant phosphopeptides, followed by identification and quantitation by liquid chromatography-tandem mass spectrometry (Figure 1). In parallel, we performed phosphoproteomic analysis of platelets activated by the strong physiological agonist thrombin and compared signaling events induced by both agonists. Using this approach, we identified 853 unique phosphorylation sites for 418 proteins in platelets. All phospho-tyrosine/serine/threonine peptides identified are listed in Table S1 in File S2. A comparison of our data and a study on platelet protein composition [26] demonstrates the capability of the approach we used to detect phosphorylation in proteins with wide range of abundance (Figure S2 in File S1 and Table S2 in File S2). Cellular localization of these proteins includes cytosol (23%), plasma membrane (21%), cytoskeleton (25%), and secretory granules (4%). Among the proteins identified, there were 46 with protein kinase activity and 12 with phosphatase activity.

**Figure 2:**
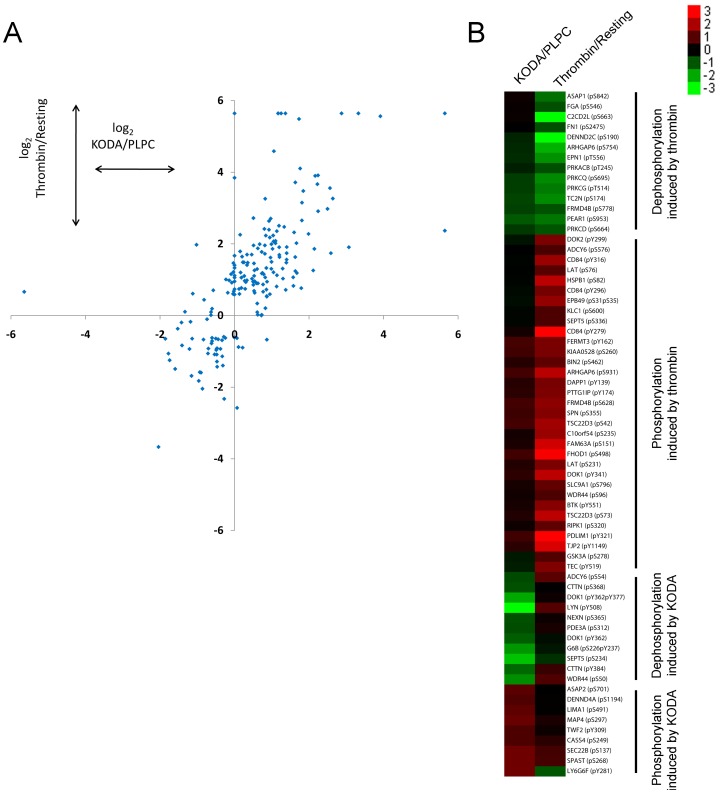
Distribution of changes in phosphorylation sites induced by KODA-PC and/or thrombin. **(A)** Common phosphorylation sites induced by KODA (x-axis; log_2_ fold change KODA/PLPC) and/or thrombin (y-axis; log_2_ fold change Thrombin/Resting). Only data with a significant change in phosphorylation induced by any agonist is shown. If peak ratio was “>10” or “<0.1” in Table S1 in File S2 (indicating a strong (de)phosphorylation) an arbitrary value of 50 (or 0.02) was assigned and the log_2_ calculated for graphical representation purpose, **(B)** Hierarchical clustering of phosphorylation sites with differences in platelets activated by KODA-PC and thrombin. Each row of the heatmap depicts an individual phosphorylation event, and each column represents the log_2_ fold change in phosphorylation induced by KODA-PC or thrombin compared to their proper control. In the heatmap, red and green represent levels of high and low phosphorylation respectively.

KODA-PC induced significant increased or decreased phosphorylation (≥1.5 fold change) of 162 sites in 115 proteins. KODA-PC induced changes are reported here for the first time. Thrombin induced significant (de)phosphorylation of 334 sites of 181 proteins. Direct comparison of phosphorylation sites found in both sets of experiments indicate that many phosphorylation events induced by KODA-PC and thrombin are similar in their trend, although the fold increase was generally higher for thrombin (Figure 2A). There were, however, notable differences at specific sites (Figure 2B). For instance, Tyr508 in LYN is dephosphorylated after platelet activation by KODA-PC, but not by thrombin. This site is relevant in the kinase function because its dephosphorylation allows for enzyme activation [27]. In contrast, KODA-PC induced phosphorylation of LY6G6F at Tyr281, while thrombin induced dephosphorylation. A previous report showed phosphorylation of this site is induced by CRP and collagen but not ADP or thrombin [28], and thus it could be an indicator for differential regulation of platelet activation by KODA-PC. There are common functional annotations for some of the proteins with at least one site differentially phosphorylated (Figure 2B). In the case of KODA-PC, we found differential phosphorylation sites on proteins associated with actin or microtubules organization (LIMA1, MAP4, TWF2, CASS4, SPAST, CTTN, and NEXN), likely reflecting changes in organization of cytoskeleton induced specifically by oxPC_CD36_. Thrombin differentially phosphorylated sites on a number of proteins containing a pleckstrin homology (PH) domain (BTK, ASAP1, FRMD4B, DOK1, DOK2, DAPP1, FERMT3, and TEC). Since recognition of phosphoinositides is a major function of PH-domains, this finding probably reflects a major role for PI-3-kinase pathway in platelets activated by thrombin as compared to KODA-PC.

Consensus motifs extracted from either phosphorylation or dephosphorylation events induced by each agonist indicated the presence of known kinase activities and binding targets (Table 1 and Figure S3 in File S1) [29], [30]. For KODA-PC, two phosphoserine motifs were enriched: RXXs (PKA and PKC substrate motifs) and sP (Pro-directed, ERK 1/2 substrate motifs, and WW domain binding motif). An additional phosphothreonine motif, PXXXt which has not been described before, was enriched in KODA-PC activated platelets. In thrombin activated platelets, in addition to RXXs and sP motifs, other motifs were present including y[E/D] (Src kinase substrate motif), RXXsL, PxsP (GSK-3, ERK 1/2, CDK5 substrate motif), and tP (WW domain binding motif). Two enriched motifs were extracted from dephosphorylation events induced by thrombin, sP and sXXN.

**Table 1:** Motifs extracted from significantly (de)phosphorylated sites induced by KODA-PC or thrombin.

KODA-PC - Phosphorylation		
Motif^1^	Known substrate/binding^2^	Proteins with motif phosphorylated
PXXXt	Unknown	G6B,TMSB4X,UBE2O,DYNC1LI1,MYL12B
RXXs	Calmodulin-dependent protein kinase II, PKA, PKC kinase substrate motif, 14-3-3 domain binding motif	ZNF185,ZDHHC5,WIPF1,TGFB1I1,TBC1D15,SYNRG,SPAST,SMTN, SLC9A1,RTN4,RASGRP2,PTPN12,PRKCD,PRKAR1A,PPP1R12A,MYLK,MYL12B,MRVI1,IQGAP2,HSPB1,FAM65C,DENND4A,DBNL,CNST, ASAP1,ARHGEF6
sP	ERK 1/2 kinase substrate motif, WW domain binding motif	ZYX,ZNF185,USP15,TGFB1I1,SLAIN2,RGC32,PDLIM1,PCBP1,MRVI1, MAVS,MAP4,LIMA1,LGALSL,INF2,HMHA1,G6B,EPB49,DNM1L,CTTN,CASS4,ASAP1
Thrombin - Phosphorylation		
Motif^1^	Known substrate/binding^2^	Proteins with motif phosphorylated
RXXs	Calmodulin-dependent protein kinase II, PKA, PKC kinase substrate motif, 14-3-3 domain binding motif	ZNF185,TBC1D15,SLC9A1,SLC9A1,SH3KBP1,SEPT5,PTPN12,PRKCD, PITPNM2,PEA15,NCK1,MYLK,MYL12B,MRVI1,HSPB1,HSPB1,FRMD4B,EPB49,EIF4G1,C10orf54,ASAP1,AMPD2,ADCY6
sP	ERK 1/2 kinase substrate motif, WW domain binding motif	ZYX,ZNF185,WIPF1,WDR44,USP15,TSC22D3,TGFB1I1,SEPT5,SAMD14,MAVS,LGALSL,KIAA0528,KALRN,FYB,FHOD1,FAM63A,EPB49, DNM1L,CTTN,CDC42BPB,BIN2,ASAP1,ARHGAP1,ADCY6
y[E/D]	Src kinase substrate motif	TPM3,SYK,STK4,PDLIM1,NCK1,INPP5D,FERMT3,DOK1,DAPP1,CTTN,CFL1,CD84,CASS4,BTK
RXXsL	Same as RXXs	WIPF1,TSC22D3,TREML1,TJAP1,TGFB1I1,TAOK1,SPN,SPAG9,RIPK1, RASGRP2,RAB7A,PLA2G4A,MYCT1,MTMR12,MRVI1,MPP1,KLC1,F2R3,DAB2,BIN2
PXsP	GSK-3, ERK 1/2, and CDK5 kinase substrate motif, WW domain binding motif	WIPF1,TSC22D3,TGFB1I1,STIM1,SAMD14,MAVS,FHOD1,FAM63A, EPB49,EPB49,DNM1L,CTTN,ASAP1,ADCY6
tP	WW domain binding motif	SPAG9,PTK2B,NBEAL2,CASS4
Thrombin – Dephosphorylation		
Motif^1^	Known substrate/binding^2^	Proteins with motif dephosphorylated
sP	ERK 1/2 kinase substrate motif, WW domain binding motif	TNIK,STK10,STIM1,SSFA2,SELP,SDPR,PEAR1,MPP1,HMHA1,ASAP1
sXXN	Unknown	SSFA2,PRKCQ,PRKCD,PRKCB,FRMD4B,DENND2C

^1^ Motif extracted using web-based software Motif-X (reference 29). Phosphorylated residues are indicated as “s”, “t”, or “y”. “X” represents any amino acid.

^2^ Assignment of substrate or binding motif based on PhosphoMotif Finder (reference 30)

We used a phosphorylation-dependent response as a gene input to identify candidate signaling components with a direct role in platelet activation by oxPC_CD36_ and thrombin by KEGG-pathway, GO-term, and Kinase Enrichment Analysis (KEA) [31] (Figure S4 in File S1 and Table S3, Table S4, and Table S5 in File S2). Most KEGG pathways identified in KODA-PC activated platelets were also observed in thrombin and point to important changes in platelet cytoskeleton. Thrombin, on the other hand, induced pathways not detectable by KODA-PC, such as MAPK signaling. As expected, GO-terms for cellular compartments confirm that upon platelet activation, many of the phosphoproteome changes take place in the plasma membrane, cytoskeleton, and cell junctions associated with an alteration in their morphology. Unique to KODA-PC was the enrichment of the GO-term “actin filament binding”, implicating specific changes in cytoskeletal organization induced by KODA-PC. Similar to KEGG-pathway analysis, KEA resulted in an overlap in inferred activated kinases between KODA-PC and thrombin, with additional kinases predicted in thrombin-induced platelets.

### Phosphoproteomic network regulating integrin activation in platelets activated by thrombin

**Figure 3:**
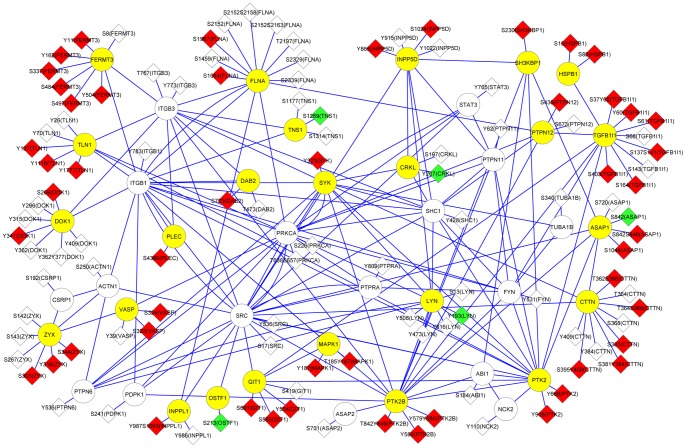
Phosphoproteome network regulating integrin in platelets activated by thrombin. Proteins and their sites of phosphorylation identified by mass spectrometry were mapped onto an integrin protein- interaction network (reference 32) as described in Materials and Methods S1. Graphical representation was done with Cytoscape. Proteins are colored based on the changes in phosphorylation (yellow circle for at least one phosphorylation event induced by thrombin; white circle for no measurable change by thrombin). Phosphorylation sites are colored based on the up-regulation (red diamond), down-regulation (green diamond), or absence of modification (white diamond) by thrombin.

We combined phosphoproteomic data from platelets activated by thrombin (Table S1 in File S2) with a protein-protein interaction database centered on integrins (integrin adhesome) [32] to gain new information on key regulatory events in platelet activation. We detected by mass spectrometry the phosphorylation of 42 out of the 157 members that constitute the integrin adhesome, and 35 of these proteins had at least one site (de)phosphorylated (≥1.5 fold change) in response to platelet activation by thrombin (Figure 3). Among the phosphoproteome changes, we observed (de)phosphorylation of proteins that directly bind to the cytoplasmic tail of integrin β3 and, directly or indirectly, regulate integrin signaling (Table 2). Among these adaptors, talin (TLN1), filamin (FLNA), and kindlin-3 (FERMT3) are key to regulate integrin inside-out signaling. Sites of phosphorylation induced by thrombin in talin are located in the FERM domain (Tyr127), vinculin binding site (Tyr1116), and rod domain (Tyr1777). The function of the two phosphorylation sites induced by thrombin in filamin is not known and one of them (Ser1967) has never been described in any cell type. Finally, nearly all observed changes in phosphorylation in kindlin-3 occurred in the FERM domain regulating the membrane localization and integrin binding.

**Table 2:** Phosphorylation dependent responses in integrin β3 adaptor proteins observed in platelets activated by thrombin.

Protein name	Gene symbol	Tryptic peptide^1^	Phosphorylation site	Fold change^2^
Kindlin-3	FERMT3	TASGDpYIDSSWELR	Y11	>10
		EKEPEEELpYDLSK	Y162	1.94
		LEGpSAPTDVLDSLTTIPELK	S337	5
		TGpSGGPGNHPHGPDASAEGLNPYGLVAPR	S484	>10
		TGSGGPGNHPHGPDApSAEGLNPYGLVAPR	S497	>10
		TGSGGPGNHPHGPDASAEGLNPpYGLVAPR	Y504	4.06
Talin	TLN1	IGITNHDEpYSLVR	Y127	2.5
		AVSSAIAQLLGEVAQGNENpYAGIAAR	Y1116	>10
		TLAESALQLLpYTAK	Y1777	>10
Filamin	FLNA	AFGPGLQGGSAGpSPAR	S1084	1.58
		VGpSAADIPINISETDLSLLTATVVPPSGR	S1967	2.4
Docking protein 1	DOK1	ADpSHEGEVAEGKLPSPPGPQELLDSPPALYAEPLDSLR	S269	1.70
		KKPLYWDLpYEHAQQQLLK	Y341	2.78
Plectin	PLEC	SSpSVGSSSSYPISPAVSR	S4386	5.04
Tyrosine-protein kinase SYK	SYK	QESTVSFNPpYEPELAPWAADK	Y323	>10
Tensin-1	TNS1	VATTPGpSPSLGR	S1269	0.61
Disabled homolog 2	DAB2	QVpSLPVTK	S723	10.16
Protein-tyrosine kinase 2-beta	PTK2B	YIEDEDpYpYKASVTR	Y579, Y580	>10
		YIEDEDYpYKASVTR	Y580	6.75
		SPLpTPEKEVGpYLEFTGPPQKPPR	T842, Y849	>10

^1^ Phosphorylation at tyrosine, serine, or threonine is indicated by “pY”, “pS”, and “pT” respectively.

^2^ Calculated as the peak area ratio of the tryptic peptide between Thrombin and Resting samples. The largest fold change is shown for peptides with the same sequence but different charge as shown in Table S1 in File S2.

### Platelet activation by KODA-PC is mediated through the SFK-SYK-PLCγ2 pathway

Closer inspection of the phosphoproteomic data from platelets activated by KODA-PC pointed to a small number of pathways that could account for an activation mechanism. Among the KEGG pathways with the highest enrichment value was Fc epsilon RI signaling, a pathway known to initiate platelet activation (Table S3 in File S2). In addition, some of the predicted activated kinases with enrichment P-values <0.05 (SYK, LYN, MYLK, and PRKG1; Table S5 in File S2) were themselves found in KODA-PC induced phosphorylation targets directly detected by mass spectrometry (Table S1 in File S2). Interestingly, there was a clear difference between KODA-PC and thrombin in the dephosphorylation of Tyr508 of LYN (Table S1 in File S2). A single point mutation at this site increases the basal level of phosphorylation in SYK and PLCγ2 in B cells [33]. We also detected a strong phosphorylation of PLCγ2, a substrate of SYK kinase, at Tyr1217 in response to KODA-PC (Table S1 in File S2). PLCγ2 synthesizes IP_3_ and diacylglycerol critical for platelet activation. Previous reports using ligands unrelated to oxPC_CD36_ have demonstrated the critical role in platelet activation of a signaling pathway, which includes the sequential activation of SFK, SYK, and PLCγ2 [34], [35]. Taken together, our bioinformatics analysis, mass spectrometry data, and previous reports on initiation of platelet activation implicate the pathway SFK-SYK-PLCγ2 as a strong candidate for follow up *in vitro* studies in KODA-PC mediated activation of platelets.

### Activation of SFK, SYK, and PLCγ2 is essential for KODA-PC induced platelet P-selectin expression

**Figure 4:**
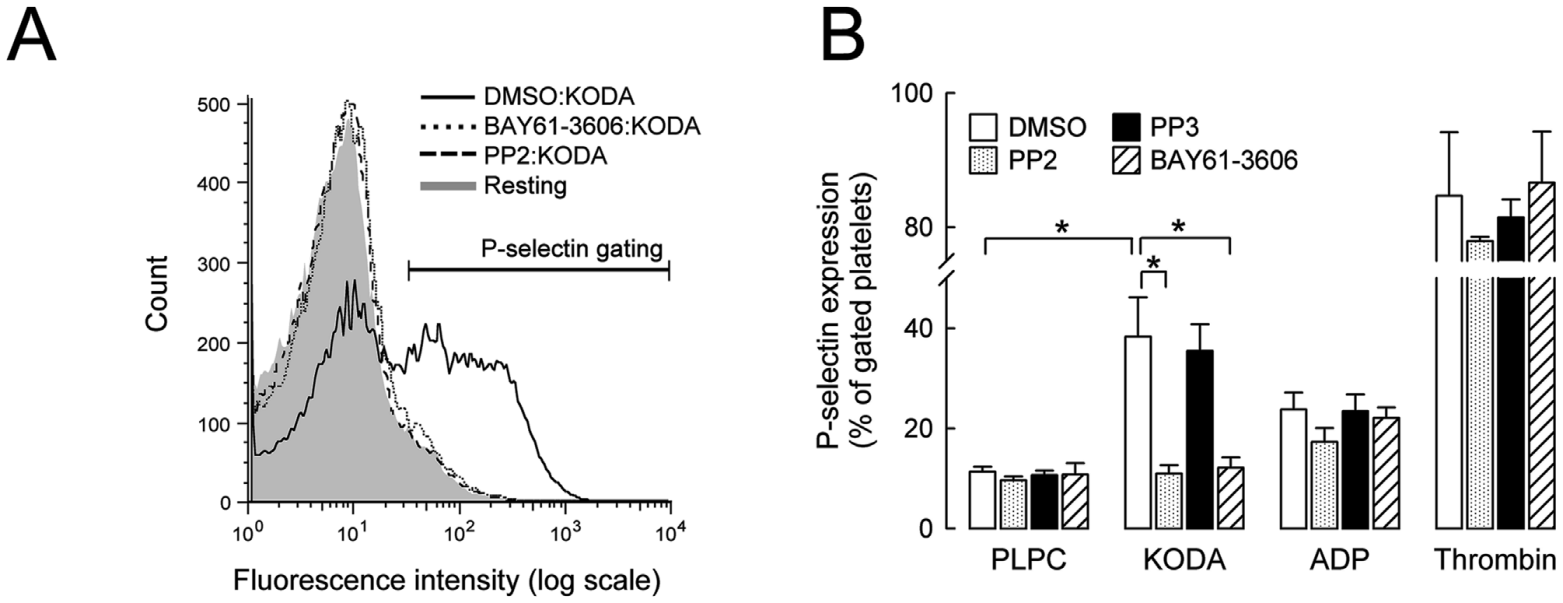
KODA-PC induces platelet P-selectin expression in a SFK- and SYK-dependent manner. **(A-B)** Human platelets isolated by gel filtration were incubated with 10 µM SFK inhibitor (PP2), 0.2 µM SYK inhibitor (BAY 61-3606), or controls (DMSO and 10 µM PP3) for 30 min. Then, 30 µM PLPC, 30 µM KODA-PC, 10 µM ADP, or 0.05 U/mL thrombin were added for 30 min. Platelet P-selectin expression was determined by flow cytometry using PE-conjugated antibody to P-selectin. **(A)** Flow cytometry histograms from representative experiments are shown. **(B)** Quantitation of flow cytometry data presented as mean ± SD of at least 3 independent experiments. * P<0.05.

We next used pharmacological inhibitors to assess the role of SFK, SYK, and PLCγ2 in platelet activation by KODA-PC. Inhibitors of SFK (PP2), SYK (BAY61-3606), and PLC (U73122) completely prevented P-selectin surface expression induced by KODA-PC in human platelets (Figures 4A, 4B, and Figure S5 in File S1). PP3, an inactive analog of PP2, had no effect. In contrast, SFK and SYK inhibitors had no effect on platelet P-selectin expression induced in platelets by weak physiological agonist ADP or strong physiological agonist thrombin, both G-protein coupled receptor agonists (Figure 4B). On the other hand, PLC inhibitor U73122 suppressed P-selectin expression induced by all the agonists tested (Figure S5 in File S1). Thus, KODA-PC, ADP, and thrombin converge on the production of the second messengers IP_3_ and DAG through different upstream signaling mechanisms. The activation of macrophages by oxPC_CD36_ depends on CD36 and the downstream target ERK, but is independent of SRC [36]. We found that ERK kinase inhibitor (PD98059) has no effect on KODA-PC induced P-selectin expression in human platelets (Figure S6 in File S1). As we showed above, platelet activation by KODA-PC is SRC dependent. Therefore, oxPC_CD36_ activate both macrophages and platelets through CD36, but the downstream signaling cascades are different. In sum, we found that signaling through SFK, SYK, and PLCγ2 is required for platelet activation by oxPC_CD36_.

### KODA-PC induces the phosphorylation of SYK and PLCγ2 via the scavenger receptor CD36 and SFK

**Figure 5:**
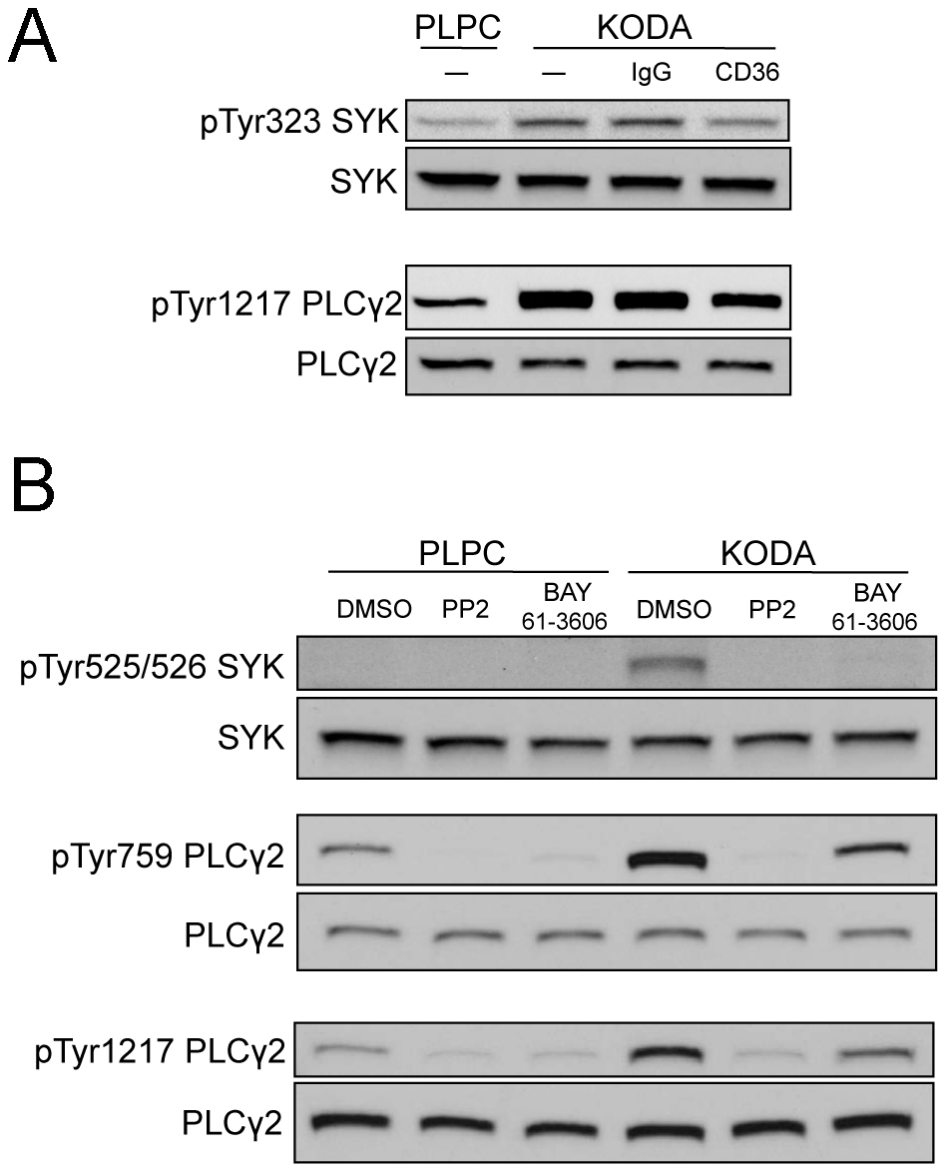
KODA-PC activates the sequential phosphorylation of SFK, SYK, and PLCγ2 through CD36. **(A)** Human platelets isolated by gel filtration were incubated with buffer alone (–), CD36 blocking antibody clone FA6-152 (CD36), or a negative control antibody (IgG); then, 50 µM PLPC or 50 µM KODA-PC were added to the platelets for 7 minutes. Equal amount of protein were separated by gel electrophoresis and probed with antibodies raised against phosphorylated SYK (pTyr323) and PLCγ2 (pTyr1217). The blots were reprobed with SYK and PLCγ2 antibodies for normalization. **(B)** Human platelets isolated by gel filtration were incubated with DMSO, 10 µM SFK inhibitor (PP2), or 0.2 µM SYK inhibitor (BAY61-3606) for 30 min; then, 50 µM PLPC or 50 µM KODA-PC were added to the platelets for 7 minutes. Equal amount of protein were separated by gel electrophoresis and probed with antibodies raised against phosphorylated SYK (pTyr525/526) and PLCγ2 (pTyr759 and pTyr1217). The blots were reprobed with SYK and PLCγ2 antibodies for normalization.

We have previously shown that oxPC_CD36_ activates platelets in a CD36 dependent manner [3]. Accordingly, we next tested whether changes in SYK and PLCγ2 phosphorylation induced by KODA-PC and detected by mass spectrometry are CD36 dependent. Anti-CD36 blocking antibody significantly suppressed the KODA-PC induced phosphorylation of SYK at Tyr323 and PLCγ2 at Tyr1217, while a negative control antibody had no effect (Figure 5A). This result confirms our mass spectrometry observations and provides evidence for the requirement of CD36 for SYK and PLCγ2 phosphorylation induced by KODA-PC in platelets.

We next studied the sequence of phosphorylation events induced by KODA-PC using specific pharmacological inhibitors. Specific platelet agonists (collagen, CRP, and rhodocytin) activate platelets through sequential activation of SFK, SYK, and PLCγ2. We investigated if the same downstream pathway takes place in platelets activated by oxidized phospholipids. KODA-PC induces strong phosphorylation of the SRC tyrosine residue (Tyr416 in SRC) (Figure S7 in File S1), an autophosphorylation event known to increase the activity of the kinase [37]. This phosphorylation is strongly inhibited by the SFK inhibitor PP2. Inhibition of SFK by PP2 prevented the phosphorylation of SYK at Tyr525/526 and PLCγ2 at Tyr759, and Tyr1217 induced by KODA-PC (Figure 5B). We also found that SYK inhibitor BAY61-3606 hinders phosphorylation of the downstream target of SYK, PLCγ2 at Tyr759 and Tyr1217 induced by KODA-PC (Figure 5B). SYK inhibitor also diminished the phosphorylation of SYK in the activation loop (Tyr525/526) (Figure 5B). These results demonstrate that activation of platelets induced by oxPC_CD36_ binding to CD36 leads to a cascade of phosphorylation initiated by SFK, followed by phosphorylation of SYK at Tyr323 and Tyr525/526. Phosphorylation of SYK is in turn followed by phosphorylation of PLCγ2 at Tyr759 and Tyr1217. Similar results were obtained with extended incubation time of platelets with KODA-PC indicating a sustained activation for up to 30 min (Figure S8 in File S1).

We tested if KODA and thrombin in combination with the chemical inhibitors of Src-kinase (PP2), SYK (BAY61-3606), or the blocking antibody to CD36 had any effect on the cell surface expression levels of CD41 (integrin α_2b_) and CD61 (integrin β_3_). We found that none of the agonist/inhibitor combinations had an effect in the levels of CD41 and CD61 in human platelets measured by flow cytometry (Figure S9 in File S1). Furthermore, KODA-PC induced changes in phosphorylation of proteins that participate in the integrin adhesome (Figure S10 in File S1) with similar trends as those found in platelet activation by thrombin. These results rule out that KODA activates platelets through an increase in cell surface expression of integrin α_2b_β_3_ or that the inhibitors work through an indirect mechanism that would decrease integrin α_2b_β_3_ cell surface expression.

## Discussion

Phosphoproteomic analysis has been successfully used for the analysis of the proteome of resting platelets, as well as for the analysis of the GP-VI mediated platelet activation [38]–[40]. However, many major pathways of platelet activation are still not investigated by this approach. In the current study, we employed a system-wide screen of protein phosphorylation by mass spectrometry to detect phosphoproteome changes induced by oxPC_CD36_, a product of phospholipid oxidation inducing platelet hyper-reactivity in hyperlipidemia, and thrombin, a strong platelet agonist. It is important to point out we performed a single experiment for each agonist and all results presented here are candidates that require further biological validation. The total number of phosphorylated proteins detected in the current study is comparable to previous phosphoproteomic studies in platelets [26], [38], [39], but comparison of the phosphorylation sites (Table S6 in File S2) shows only 256 sites overlap between our study and the most extensive study on platelet phosphoproteomics [26]. This may be an indication that our phosphoproteomic screen is biased towards the most abundant phosphopeptides when platelets are activated by an agonist. Information on changes in phosphorylation levels at specific sites induced by agonists is less common at the moment [40] due to the challenges of agonist-platelet experiments with large scale sampling. The analysis of our data revealed numerous phosphorylation events that occurred in proteins whose platelet-related function has just recently been recognized or is not known indicating the discovery aspect of the approach and the relevance of the results. For example, both agonists studied induced significant changes in phosphorylation sites of SEPT5 and FERMT3, and deficiency of these proteins causes bleeding disorders associated with platelet defects [41], [42]. Also, KODA-PC induced dephosphorylation of LRRFIP1 at Ser115, a protein with variations in human platelets that affects platelet reactivity and is associated with myocardial infarction [43]. Both agonists induced strong phosphorylation changes in proteins with unknown function in platelets such as HMHA1 (Minor histocompatibility protein HA-1), LGALSL (Galectin-related protein), or ZNF185 (Zinc finger protein 185).

The current study provides new information on 334 phosphorylation sites of 181 proteins induced in platelets activated by thrombin. Many phosphorylation responses and pathways inferred from the bioinformatic analysis are in agreement with known pathways activated by thrombin, such as changes in cell shape, protein kinase C, or MAP kinase activation [44]. We focused on the phosphoproteome network associated with integrin activation because of its critical function in platelets and the absence of comprehensive data on site-specific phosphorylation induced by thrombin or other platelet agonists. One of the informative findings was changes in phosphorylation status of multiple sites of talin, filamin, and kindlin-3. Changes in these sites are likely to induce conformational changes of the proteins or their binding to target regulators, thereby contributing to the ability of these proteins to regulate integrin α_2b_β_3_ [45]. We also identified a large number of Src-kinase substrates through Motif-X that are phosphorylated after activation of platelets by thrombin. These phosphorylation events do not participate in the initial activation by thrombin (Src-kinase inhibitor PP2 did not block platelet activation by thrombin) but may be important in later stages of platelet regulation and aggregation.

Platelet activation by KODA-PC was used as a model for the activation of platelets by pro-thrombotic oxidized phospholipids. We identified changes in protein phosphorylation of 115 proteins in platelets stimulated by KODA-PC. One of the consensus motifs (PXXXt) extracted from upregulated phosphorylation events has not been previously reported in the literature. The closest reference is a motif (PXXX[t/s]XXC) present in peroxiredoxins with antioxidant properties [46], but none of the proteins detected in our results contained the Cys at +3 associated with the enzymatic activity. From our phosphoproteomic/bioinformatic analysis, we identified a candidate mechanism for platelet activation by oxidized phospholipids that includes SFK, SYK, and PLCγ2. *Ex vivo* validation experiments (Figure 4 and Figure 5) confirmed that the SFK-SYK-PLCγ2 pathway is downstream of the scavenger receptor CD36 and functions as a signaling axis for platelet activation by oxPC_CD36_.

Comparison of our mass spectrometry results (Table S1 in File S2) to previous reports on platelet activation by collagen and regulation by SYK allows us to expand the signaling network beyond the core pathway formed by SFK, SYK, and PLCγ2. The proteins previously described in platelet activation include FCER1G [35], LY6G6F [28], FLNA [47], G6B [48], FES [49], PRKCQ [50], OSTF1 [28], and PTPRJ [51]. FCER1G is particularly interesting because it contains an ITAM-motif that allows SYK binding and activation [52]. Other phosphorylation events relate to inhibitory mechanisms that may prevent strong platelet activation by KODA-PC. For instance, KODA-PC induces phosphorylation of PKCδ (PRKCD) at Ser304 and Tyr313, a protein that prevents granule secretion by collagen [53]. Phosphorylation of PKCδ at Tyr313 (indicated as Tyr311 in many publications) is important for its activity [54]. Rottlerin, an inhibitor to PKCδ, enhanced P-selectin expression in human platelets induced by KODA-PC (data not shown), suggesting that PKCδ indeed down-regulates platelet activation by oxidized phospholipids. Similarly, MRVI1 (or IRAG) is involved in platelet regulation, and mouse models lacking this protein have increased platelet aggregation in response to thrombin and collagen [55].

Platelet shape change, a critical part of platelet activation, depends on actin polymerization leading to the formation of a variety of higher order cytoplasmic structures involved into the formation of filopodia, lamellipodia and eventual spreading on the surface of adhesive substrate. A large number of actin-binding proteins regulate actin assembly and disassembly. Interestingly, we observed that KODA significantly and specifically regulates a number of actin binding proteins known to play a role in thrombosis. These include filamin A, supervillin, and non-muscle myosin heavy chain 9. Filamin A, an actin binding protein crosslinking F-actin fibers, is known to bind to the cytoplasmic tail of β-integrins and to coordinate the interaction between integrins and the cytoskeleton. Alterations in platelet function in patients with filamin A mutations are reported [56]. Cytoskeletal regulatory protein supervillin may serve as a high-affinity link between the actin cytoskeleton and the membrane. Its expression negatively regulates platelet reactivity and thrombus formation [57]. Non-muscle myosin heavy chain 9 plays a key role in platelet contractile phenomena and outside-in signaling [58]. Several actin binding proteins with a function not yet demonstrated in platelets are also affected by KODA. These include nexilin, which binds to F-actin and may function in cell adhesion adhesion, derbin-like protein, which participates in lamellipodia formation, dystonin, a protein connecting intermediate filaments to actin, and LIMA1 (eplin), an inhibitor of actin filament depolymerization. Taken together, these data suggest that specific oxidized phospholipids ligands for CD36 induce a disproportionally strong effect on platelet cytoskeleton.

Throughout this paper, we have described the role of Src family kinases (SFK) without assigning the specific member(s) of the family. Western blotting analysis detected an increase in phosphorylation of several bands corresponding to members of the SFK (Figure S7 in File S1). LYN and FYN, members of the SFK, associate to CD36 when platelets are activated by oxLDL, a lipoprotein containing oxidized phospholipids [12]. Even though LYN is the most important SFK in platelet activation by agonists such as collagen, double knockout combinations of LYN and another SFK are more effective to delay platelet activation and inhibit FCER1G phosphorylation than single knock out of LYN in mice [59]. Therefore, it seems reasonable to hypothesize overlapping roles for members of the SFK in initiating platelet activation by oxPC_CD36_ that will need to be elucidated in future studies.

Previous reports demonstrated that CD36 is required for induction of apoptosis in macrophages by oxidized phospholipids [36]. Activation in macrophages requires ERK and is independent of SFK. In contrast, the activation of platelets by oxPC_CD36_ requires CD36 and SFK but it is independent of ERK (Figure S6 in File S1). In aortic endothelial cells, both SFK and ERK are key in the signaling cascade induced by oxidized products of PAPC [22], [60]. Lack of CD36 participation in aortic endothelial cell activation by oxidized PAPC [61] further extends the divergence in signal mechanisms elicited by the same ligands in endothelial cells, platelets, and macrophages, representing different cell types with prominent roles in cardiovascular disease. These differences can help guide specifically targeted kinase inhibitor-based therapeutic approaches. Taken together, our results provide insights into the mechanism of platelet activation by oxidized phospholipids, compounds generated in conditions of hyperlipidemia and oxidative stress and modulating platelet function *in vivo*.

## Supporting Information

File S1 **Figure S1.** KODA-PC induces the phosphorylation of numerous proteins in human platelets. **Figure S2.** Abundance of proteins identified by phosphoproteomics in platelets based on Burkhart et al (Blood, 120: e73-82, 2012). **Figure S3.** Motif-X analysis of sites that were (de)phosphorylated after platelet activation by KODA-PC and thrombin. **Figure S4.** Selected GO-terms enriched from the list of genes identified by phosphoproteomic in platelets activated by KODA-PC and thrombin. **Figure S5.** PLC inhibitor blocks platelet P-selectin expression induced by different ligands. **Figure S6.** ERK kinase is not required for P-selectin expression induced by KODA-PC. **Figure S7.** KODA-PC induces Src-family kinases (SFK) phosphorylation. **Figure S8.** SYK and PLCγ2 phosphorylation induced by KODA-PC at different time points in the presence of CD36 blocking antibody and chemical inhibitors to SFK and SYK. **Figure S9.** Surface level expression of CD41 and CD61 is not affected by KODA-PC, thrombin, chemical inhibitors PP2 and BAY61-3606, or blocking antibody to CD36. **Figure S10.** Phosphoproteome network regulating integrin in platelets activated by KODA-PC.
(PDF)

File S2 **Table S1.** Identification and quantitation of protein phosphorylation sites from platelets activated with KODA and thrombin using enrichment of phosphopeptides. **Table S2.** Number of copies and rank of phosphorylated proteins detected in this study. **Table S3.** Complete list of KEGG pathways enriched in the list of phosphoproteins modulated by KODA and thrombin in platelets. **Table S4.** Complete list of GO-terms enriched in the list of phosphoproteins modulated by KODA and thrombin in platelets. **Table S5.** Prediction of kinases responsible for KODA- and Thrombin-induced protein phosphorylation. **Table S6.** List of shared phosphoryaltion sites described in Table S1 and Burkhart, J.M. et al. Blood. 120: e73-82, 2012.
(XLSX)

Materials and Methods S1 **Detailed description of trypsin digestion of the protein lysate, phosphopeptide enrichment, mass spectrometry analysis, chromatography alignment, quantitation, and bioinformatics and enrichment analysis employed to study the phosphoproteome changes induced by the agonists.**
(PDF)

Annotated Spectra S1 (ZIP)

Annotated Spectra S2 (ZIP)

Annotated Spectra S3 (ZIP)

Annotated Spectra S4 (ZIP)

Annotated Spectra S5 (ZIP)
