# Supplementary material for: Phosphoproteomic Analysis of Platelets Activated by Pro-Thrombotic Oxidized Phospholipids and Thrombin
Source: PLoS One. 2014 Jan 6;9(1):e84488. doi: 10.1371/journal.pone.0084488 (PMC3882224; doi:10.1371/journal.pone.0084488)
Supplement: File S1 — Figure S1. KODA-PC induces the phosphorylation of numerous proteins in human platelets. Figure S2. Abundance of proteins identified by phosphoproteomics in platelets based on Burkhart et al (Blood, 120: e73-82, 2012). Figure S3. Motif-X analysis of sites that were (de)phosphorylated after platelet activation by KODA-PC and thrombin. Figure S4. Selected GO-terms enriched from the list of genes identified by phosphoproteomic in platelets activated by KODA-PC and thrombin. Figure S5. PLC inhibitor blocks platelet P-selectin expression induced by different ligands. Figure S6. ERK kinase is not required for P-selectin expression induced by KODA-PC. Figure S7. KODA-PC induces Src-family kinases (SFK) phosphorylation. Figure S8. SYK and PLCγ2 phosphorylation induced by KODA-PC at different time points in the presence of CD36 blocking antibody and chemical inhibitors to SFK and SYK. Figure S9. Surface level expression of CD41 and CD61 is not affected by KODA-PC, thrombin, chemical inhibitors PP2 and BAY61-3606, or blocking antibody to CD36. Figure S10. Phosphoproteome network regulating integrin in platelets activated by KODA-PC. (PDF) [file pone.0084488.s001.pdf]

FIGURES S1 – S10

Figure S1. KODA-PC induces the phosphorylation of numerous proteins in human platelets. Human platelets isolated by gel filtration were incubated with 50 µM PLPC or 50 µM KODA-PC for 5 and 20 minutes. Equal amount of protein were separated by gel electrophoresis and probed with antibodies raised against (**A**) phospho-tyrosine (Cell Signaling, catalog # 9411) and (**B**) phospho-Ser PKC substrate (Cell Signaling, catalog # 2261) as indicators of changes in tyrosine, serine, and threonine phosphorylation.

Figure S2. Abundance of proteins identified by phosphoproteomics in platelets based on Burkhart et al (Blood, 120: e73-82, 2012). Phosphorylated proteins identified in this study were matched with protein abundance in platelets as reported by Burkhart et al (Blood, 120: e73-82, 2012). Unique proteins from the original reference or proteins identified in our study were grouped based on their abundance (estimated copies/platelet) and the percentage of each group calculated.

Figure S3. Motif-X analysis of sites that were (de)phosphorylated after platelet activation by KODA-PC and thrombin. The tryptic peptides identified by phosphoproteomics were grouped based on phosphorylation and dephosphorylation changes and used for motif extraction with Motif-X as described in Materials and Methods S1. Sequence logos generated by Motif-X software and the predicted substrate/ binding motifs are shown.

Figure S4. Selected GO-terms enriched from the list of genes identified by phosphoproteomic in platelets activated by KODA-PC and thrombin. The list of proteins identified by phosphoproteomics (all) and proteins that changed in phosphorylation for the indicated agonist (changed) were analyzed for GO-term enrichment in **(A)** cellular component, **(B)** biological process, and **(C)** molecular function. Only selected terms are shown in the graphs. The complete list of terms and enrichment values is shown in Table S4 in File S2.

Figure S5. PLC inhibitor blocks platelet P-selectin expression induced by different ligands. Human platelets isolated by gel filtration were incubated with 10 µM PLC inhibitor (U73122), or control (DMSO) for 30 min. Then, 50 µM KODA-PC, 10 µM ADP, or 0.05 U/mL thrombin were added for 30 min. Platelet P-selectin expression was determined using PE-conjugated antibody to P-selectin.

Figure S6. ERK kinase is not required for P-selectin expression induced by KODA-PC. Human platelets isolated by gel filtration were incubated with 10 µM SFK inhibitor (PP2), 10 µM ERK kinase inhibitor (PD98059), or control (DMSO) for 30 min. Then, 30 µM KODA-PC was added for 30 min. Platelet P-selectin expression was determined using PE-conjugated antibody to P-selectin. (**A**) Flow

cytometry histograms from representative experiments are shown. (**B**) Quantitation of flow cytometry data presented as mean ± SD of at least 3 independent experiments. * $P < 0.05$.

Figure S7. KODA-PC induces Src-family kinases (SFK) phosphorylation. Human platelets isolated by gel filtration were incubated with DMSO or 10 μM SFK inhibitor (PP2); then, 50 μM PLPC or 50 μM KODA-PC were added to the platelets for 7 minutes. Equal amount of protein were separated by gel electrophoresis and probed with antibodies raised against phosphorylated SFK (pTyr416).

Figure S8. SYK and PLCγ2 phosphorylation induced by KODA-PC at different time points in the presence of CD36 blocking antibody and chemical inhibitors to SFK and SYK. (A) Human platelets isolated by gel filtration were incubated with CD36 blocking antibody clone FA6-152 (CD36), or a negative control antibody (IgG); then, 50 μM PLPC or 50 μM KODA-PC were added to the platelets for 5 and 30 minutes. Equal amount of protein were separated by gel electrophoresis and probed with antibodies raised against phosphorylated SYK (pTyr525/526) and PLCγ2 (pTyr1217). The blots were reprobed with SYK and PLCγ2 antibodies for normalization. **(B)** Human platelets isolated by gel filtration were incubated with DMSO, 10 μM SFK inhibitor (PP2), or 0.2 μM SYK inhibitor (BAY61-3606) for 30 min; then, 50 μM PLPC or 50 μM KODA-PC were added to the platelets for 5 and 30 minutes. Equal amount of protein were separated by gel electrophoresis and probed with antibodies raised against phosphorylated SYK (pTyr525/526) and PLCγ2 (pTyr1217). The blots were reprobed with SYK and PLCγ2 antibodies for normalization.

Figure S9. Surface level expression of CD41 and CD61 is not affected by KODA-PC, thrombin, chemical inhibitors PP2 and BAY61-3606, or blocking antibody to CD36. Human platelets isolated by gel filtration were incubated with 10 μM SFK inhibitor (PP2), 0.2 μM SYK inhibitor (BAY 61-3606), blocking antibody to CD36 (CD36), or controls (DMSO or IgG) for 30 min. Then, 50 μM KODA-PC, 0.05 U/mL thrombin, or buffer alone (Tyrode's buffer) were added for 30 min. (**A**) Platelet CD41 (integrin α_{2b}) expression was determined by flow cytometry using PE-conjugated antibody to CD41 (Millipore, catalog # MAB1207). (**B**) Platelet CD61 (integrin β_3) expression was determined by flow cytometry using PE-conjugated antibody to CD61 (BD Biosciences, catalog # 555754). (**A, B**) PE-conjugated mouse IgG_1 (PE-IgG, BD Biosciences, catalog # 555749) was used for control.

Figure S10. Phosphoproteome network regulating integrin in platelets activated by KODA-PC. Proteins and their sites of phosphorylation identified by mass spectrometry were mapped onto an integrin protein-interaction network (reference 32) as described in Materials and Methods. Graphical representation was done with Cytoscape. Proteins are colored based on the changes in phosphorylation (yellow circle for at least one phosphorylation event induced by thrombin; white circle for no measurable

change by thrombin). Phosphorylation sites are colored based on the up-regulation (red diamond), down-regulation (green diamond), or absence of modification (white diamond) by KODA-PC.

Figure S1

A

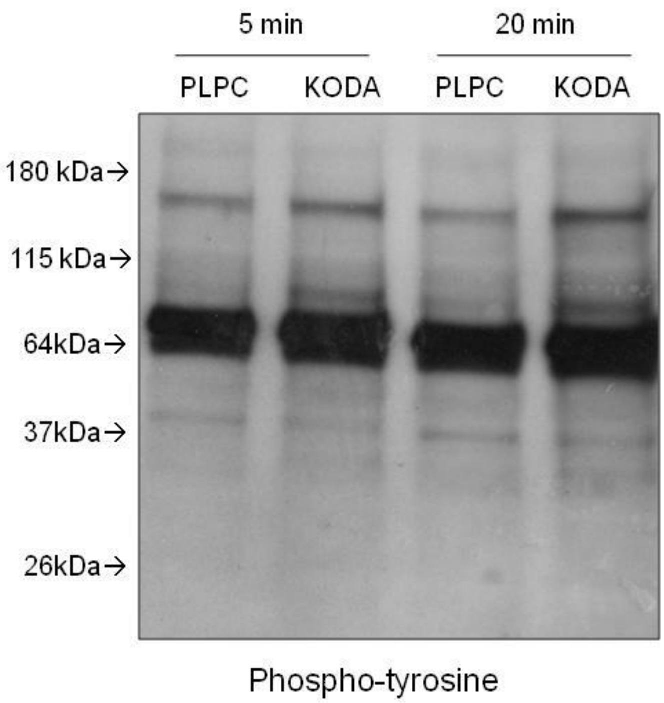
5 min
20 min
PLPC
KODA
PLPC
KODA
180 kDa→
115 kDa→
64kDa→
37kDa→
26kDa→
Phospho-tyrosine

B

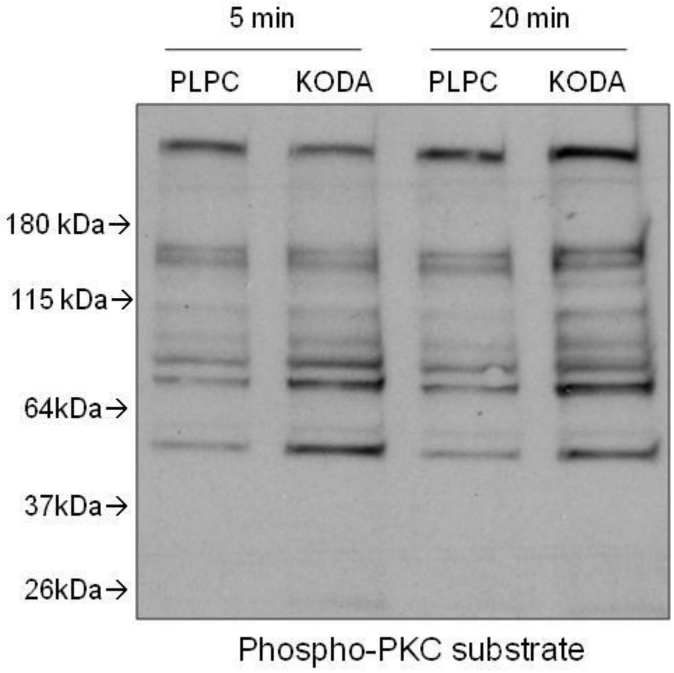
5 min
20 min
PLPC
KODA
PLPC
KODA
180 kDa→
115 kDa→
64kDa→
37kDa→
26kDa→
Phospho-PKC substrate

Figure S2

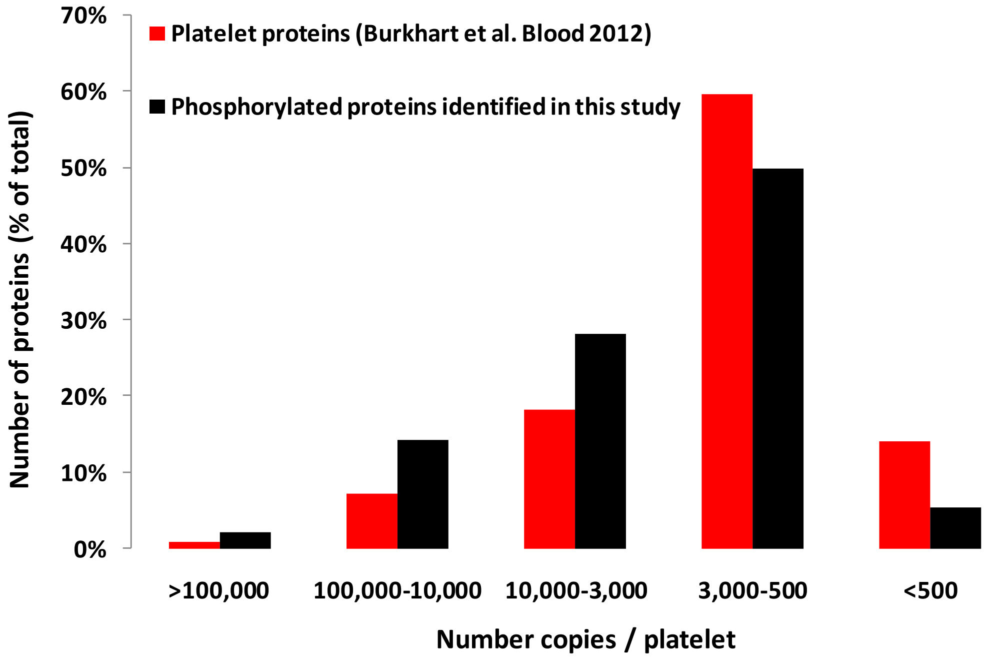

70%
60%
50%
40%
30%
20%
10%
0%
Number of proteins (% of total)
Platelet proteins (Burkhart et al. Blood 2012)
Phosphorylated proteins identified in this study
>100,000
100,000-10,000
10,000-3,000
3,000-500
<500
Number copies / platelet

Figure S3

<u>**KODA-induced phosphorylation**</u>	<u>**Substrate/binding motif**</u>
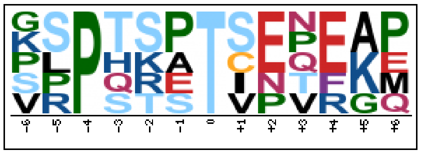	**Unknown**
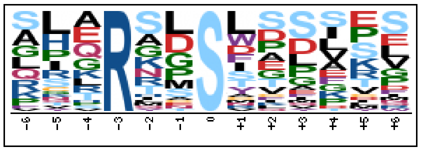	**Calmodulin-dependent protein kinase II, PKA, PKC substrate motif 14-3-3 domain binding motif**
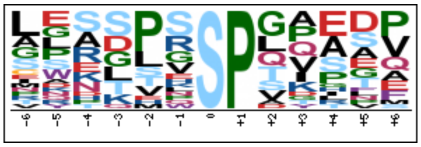	**ERK1, ERK2 Kinase substrate motif WW domain binding motif**

<u>**Thrombin-induced dephosphorylation**</u>	<u>**Substrate/binding motif**</u>
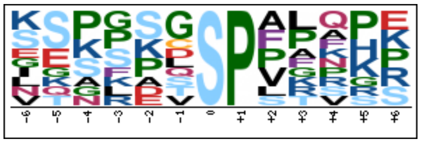	**ERK1, ERK2 Kinase substrate motif WW domain binding motif**
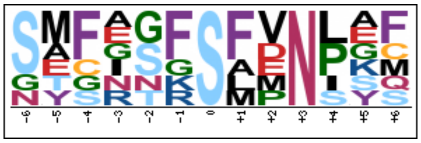	**Unknown**

Thrombin-induced phosphorylation	Substrate/binding motif
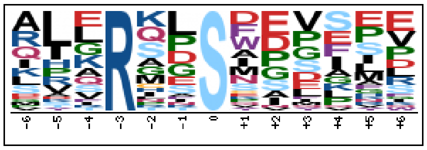	**Calmodulin-dependent protein kinase II,<br>PKA, PKC substrate motif<br>14-3-3 domain binding motif**
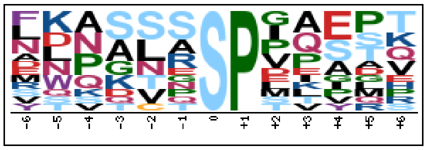	**ERK1, ERK2 Kinase substrate motif<br>WW domain binding motif**
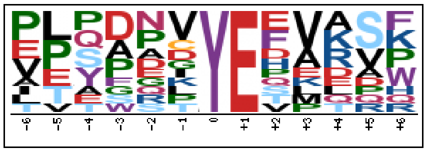	**Src kinase substrate motif**
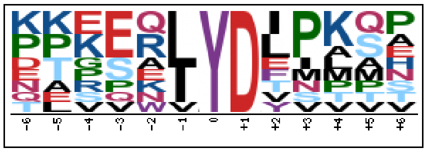	**Src kinase substrate motif**
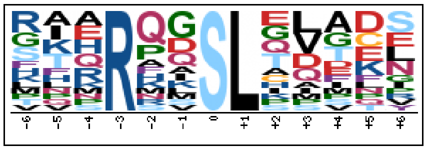	**Calmodulin-dependent protein kinase II,<br>PKA, PKC substrate motif<br>14-3-3 domain binding motif**
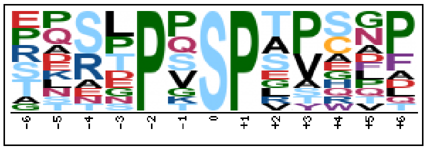	**GSK, ERK1, ERK2, CDK5 substrate motif<br>WW domain binding motif**
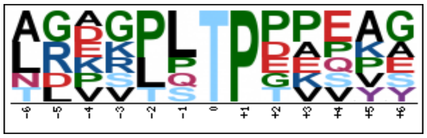	**WW domain binding motif**

Figure S4

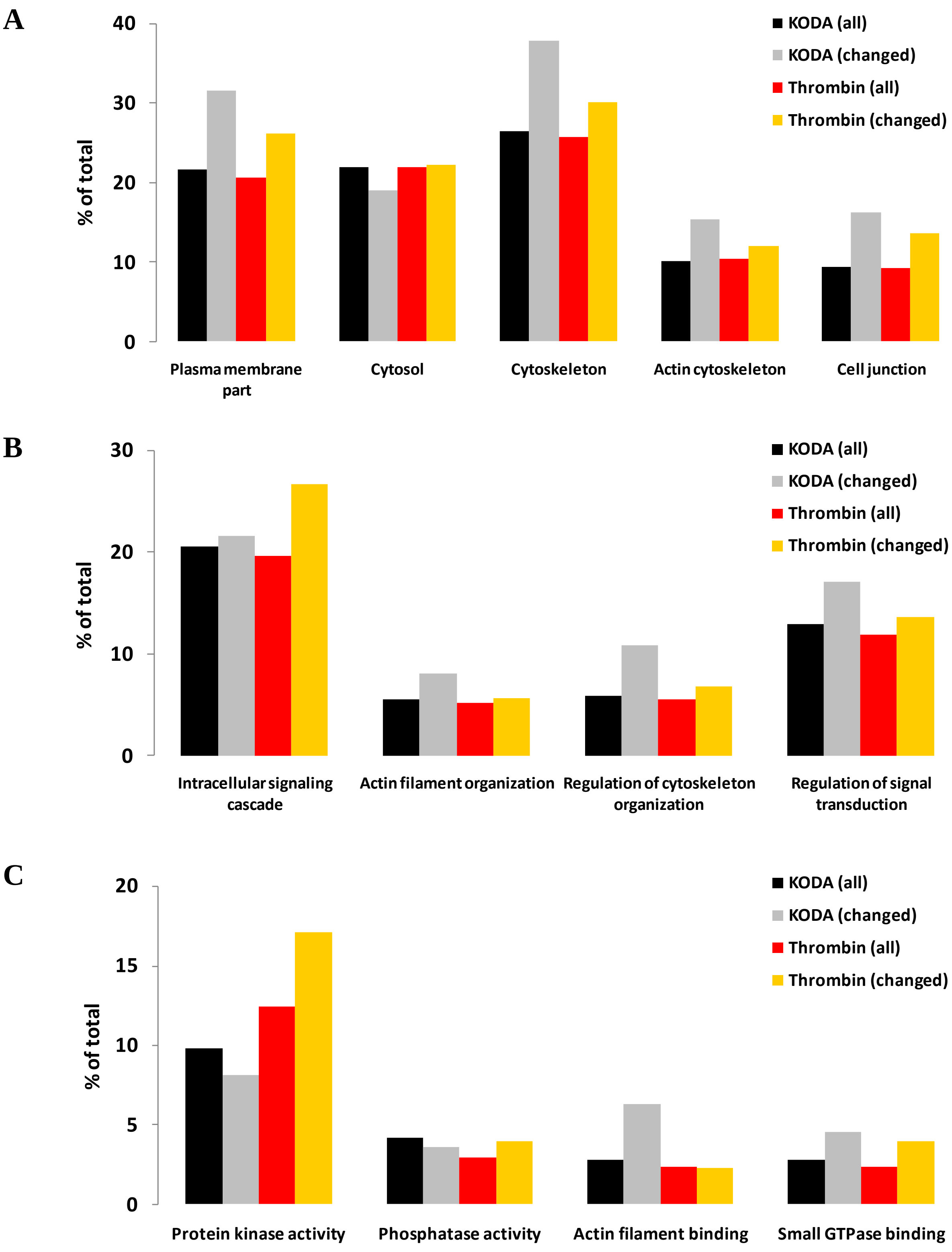
A
40
30
20
10
0
% of total
KODA (all)
KODA (changed)
Thrombin (all)
Thrombin (changed)
Plasma membrane part
Cytosol
Cytoskeleton
Actin cytoskeleton
Cell junction
B
30
20
10
0
% of total
KODA (all)
KODA (changed)
Thrombin (all)
Thrombin (changed)
Intracellular signaling cascade
Actin filament organization
Regulation of cytoskeleton organization
Regulation of signal transduction
C
20
15
10
5
0
% of total
KODA (all)
KODA (changed)
Thrombin (all)
Thrombin (changed)
Protein kinase activity
Phosphatase activity
Actin filament binding
Small GTPase binding

Figure S5

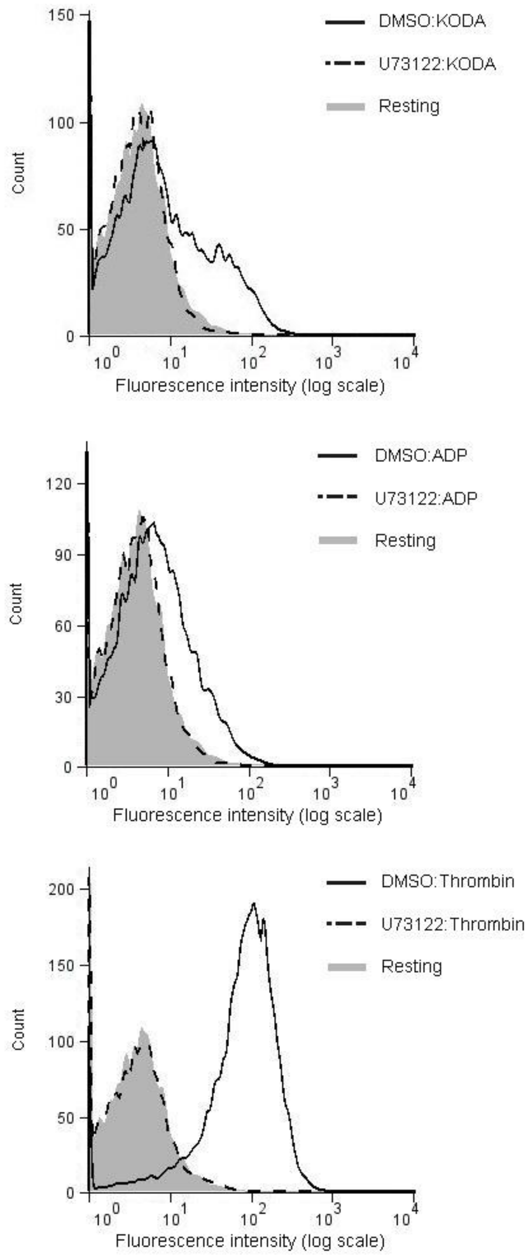
DMSO:KODA
U73122:KODA
Resting
Count
Fluorescence intensity (log scale)
DMSO:ADP
U73122:ADP
Resting
Count
Fluorescence intensity (log scale)
DMSO:Thrombin
U73122:Thrombin
Resting
Count
Fluorescence intensity (log scale)

Figure S6

A

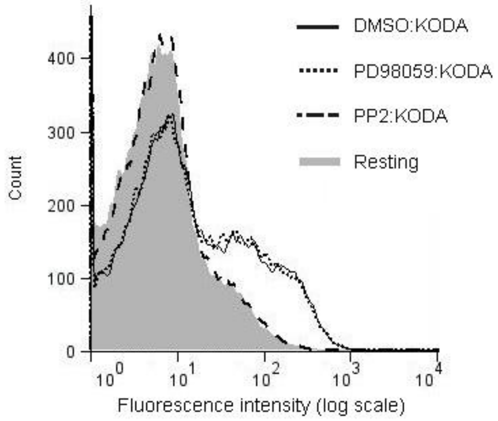
DMSO:KODA
PD98059:KODA
PP2:KODA
Resting
Count
400
300
200
100
0
Fluorescence intensity (log scale)

B

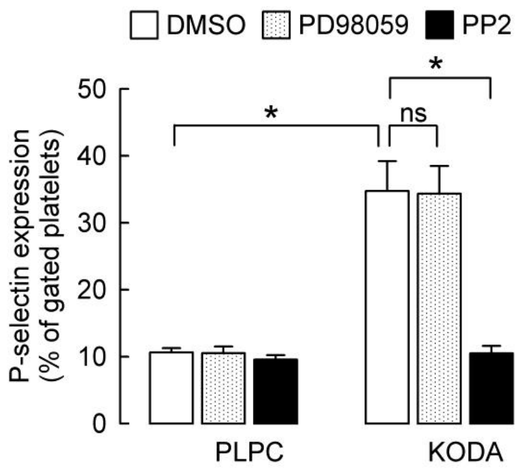
DMSO
PD98059
PP2
P-selectin expression
(% of gated platelets)
50
40
30
20
10
0
*
ns
*
PLPC
KODA

Figure S7

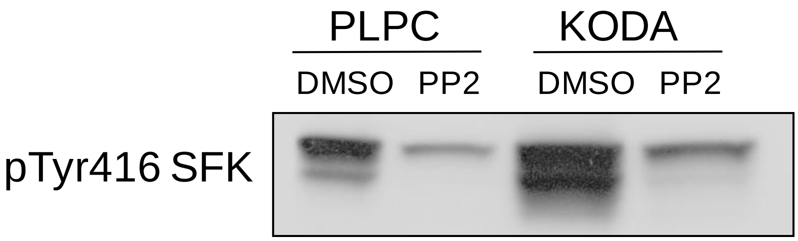

Figure S8

A

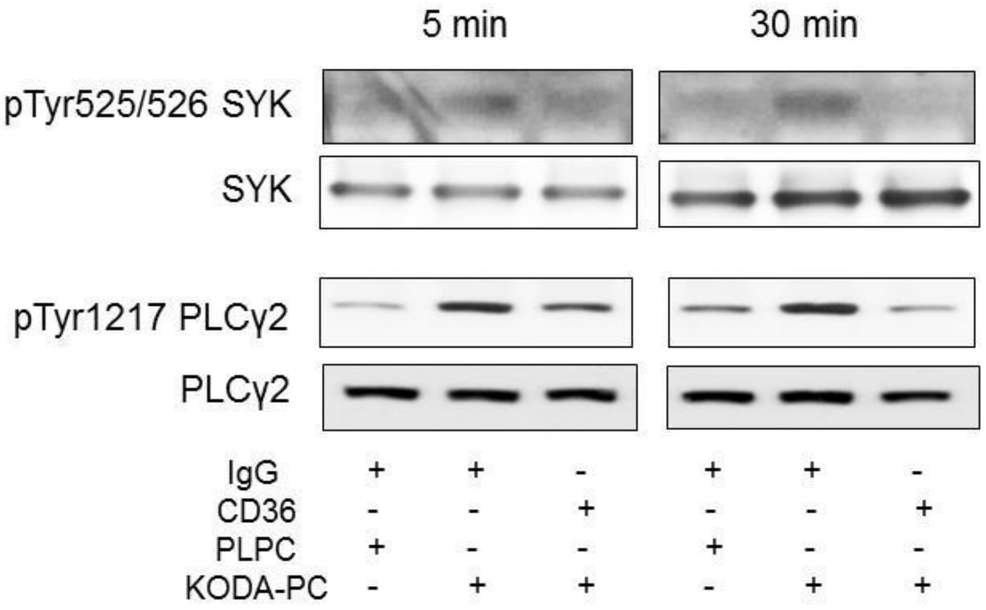
5 min
30 min
pTyr525/526 SYK
SYK
pTyr1217 PLCγ2
PLCγ2
IgG + + - + + -
CD36 - - + - - +
PLPC + - - + - -
KODA-PC - + + - + +

B

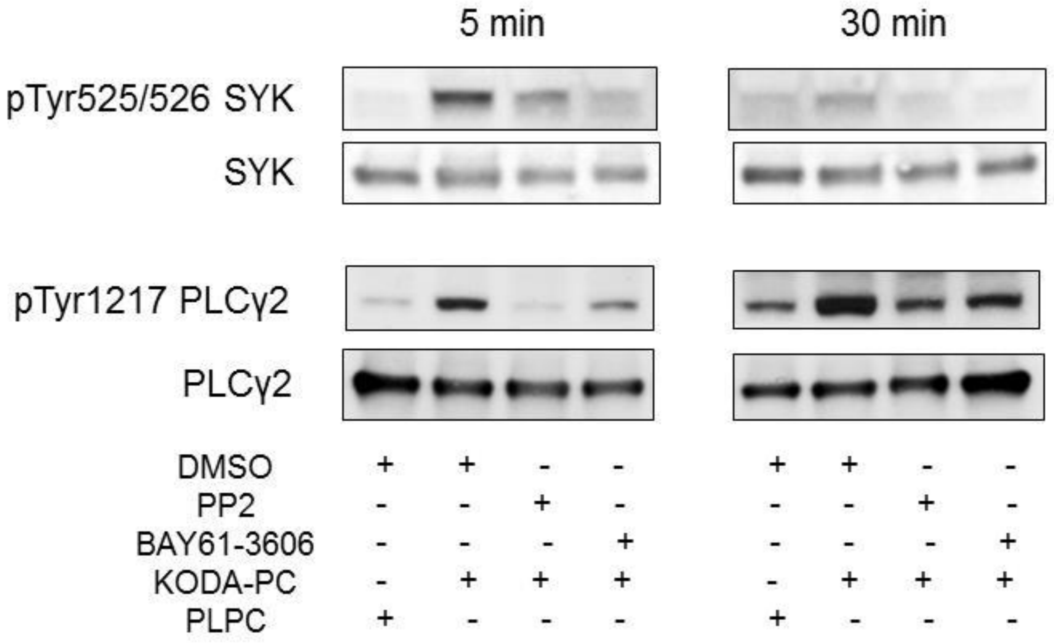
5 min
30 min
pTyr525/526 SYK
SYK
pTyr1217 PLCγ2
PLCγ2
DMSO + + - - + + - -
PP2 - - + - - - + -
BAY61-3606 - - - + - - - +
KODA-PC - + + + - + + +
PLPC + - - - + - - -

Figure S9

A

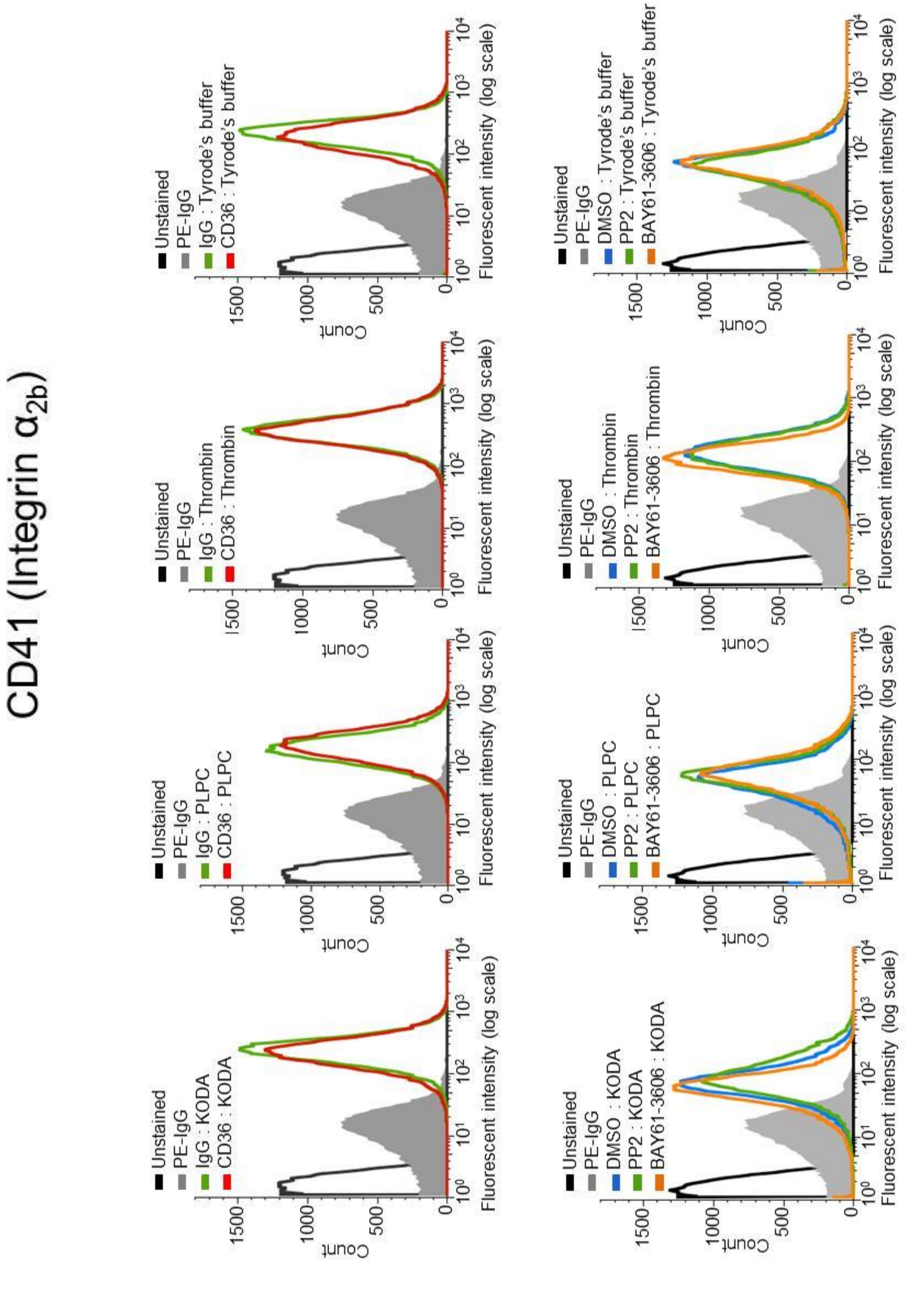

CD41 (Integrin α2b)
Unstained
PE-IgG
IgG : KODA
CD36 : KODA
IgG : PLPC
CD36 : PLPC
IgG : Thrombin
CD36 : Thrombin
IgG : Tyrode's buffer
CD36 : Tyrode's buffer
DMSO : KODA
PP2 : KODA
BAY61-3606 : KODA
DMSO : PLPC
PP2 : PLPC
BAY61-3606 : PLPC
DMSO : Thrombin
PP2 : Thrombin
BAY61-3606 : Thrombin
DMSO : Tyrode's buffer
PP2 : Tyrode's buffer
BAY61-3606 : Tyrode's buffer
Count
Fluorescent intensity (log scale)

B

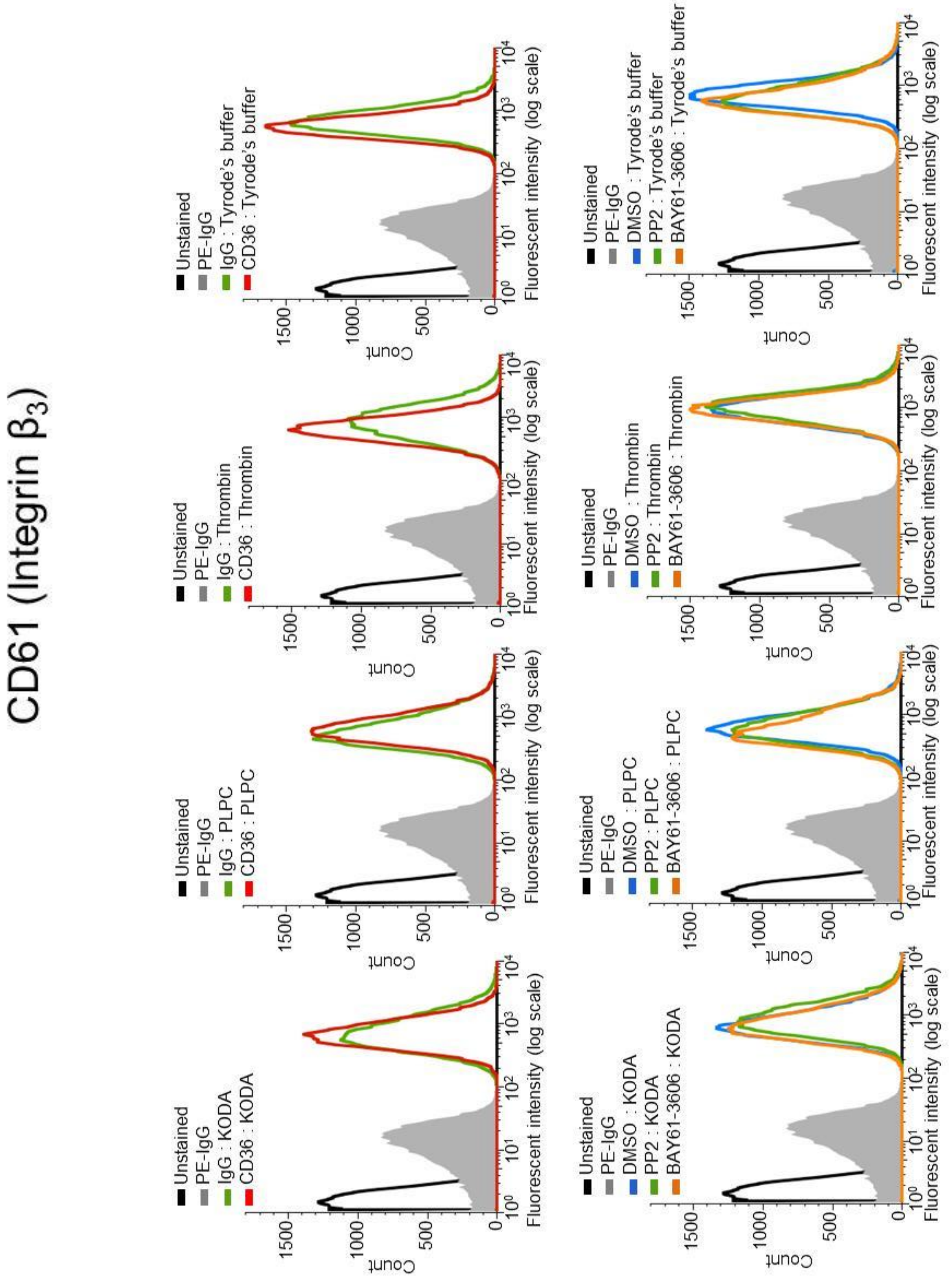
CD61 (Integrin β3)
Unstained
PE-IgG
IgG : KODA
CD36 : KODA
IgG : PLPC
CD36 : PLPC
IgG : Thrombin
CD36 : Thrombin
IgG : Tyrode's buffer
CD36 : Tyrode's buffer
DMSO : KODA
PP2 : KODA
BAY61-3606 : KODA
DMSO : PLPC
PP2 : PLPC
BAY61-3606 : PLPC
DMSO : Thrombin
PP2 : Thrombin
BAY61-3606 : Thrombin
DMSO : Tyrode's buffer
PP2 : Tyrode's buffer
BAY61-3606 : Tyrode's buffer
Count
Fluorescent intensity (log scale)

Figure S10

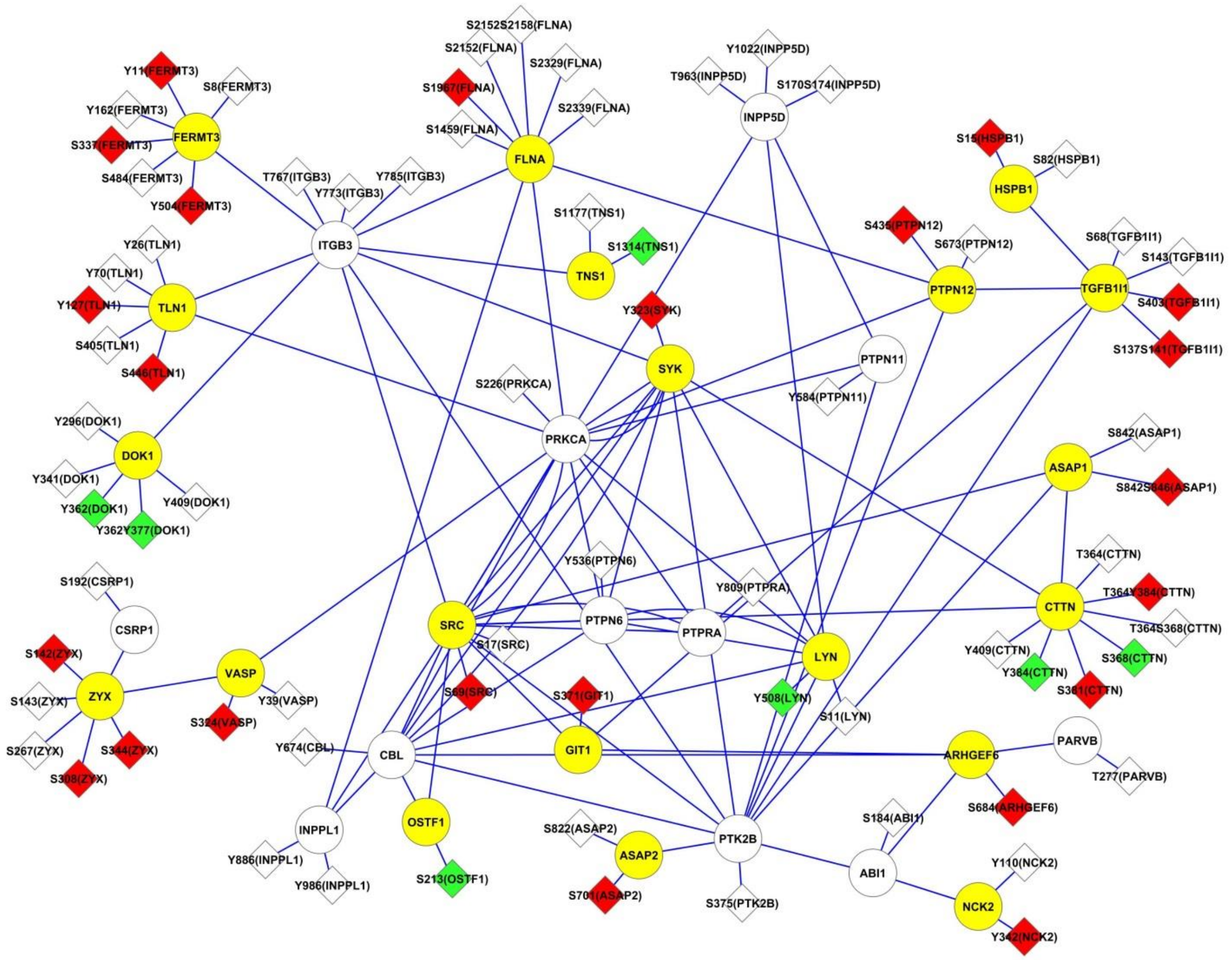
S2152S2158(FLNA)
S2152(FLNA)
S2329(FLNA)
S1967(FLNA)
S2339(FLNA)
S1459(FLNA)
FLNA
Y1022(INPP5D)
T963(INPP5D)
S170S174(INPP5D)
INPP5D
Y11(FERMT3)
S8(FERMT3)
Y162(FERMT3)
S337(FERMT3)
FERMT3
S484(FERMT3)
Y504(FERMT3)
T767(ITGB3)
Y785(ITGB3)
Y773(ITGB3)
ITGB3
S15(HSPB1)
S82(HSPB1)
HSPB1
S1177(TNS1)
S1314(TNS1)
TNS1
S435(PTPN12)
S673(PTPN12)
PTPN12
S68(TGFB1I1)
S143(TGFB1I1)
TGFB1I1
S403(TGFB1I1)
S137S141(TGFB1I1)
Y26(TLN1)
Y70(TLN1)
Y127(TLN1)
TLN1
S405(TLN1)
S446(TLN1)
Y323(SYK)
SYK
PTPN11
Y584(PTPN11)
S226(PRKCA)
PRKCA
Y296(DOK1)
DOK1
Y341(DOK1)
Y362(DOK1)
Y409(DOK1)
Y362Y377(DOK1)
S842(ASAP1)
ASAP1
S842S846(ASAP1)
Y536(PTPN6)
T364(CTTN)
T364Y384(CTTN)
CTTN
T364S368(CTTN)
Y809(PTPRA)
S192(CSRP1)
CSRP1
S142(ZYX)
ZYX
S143(ZYX)
S267(ZYX)
S344(ZYX)
S308(ZYX)
VASP
S324(VASP)
Y39(VASP)
SRC
S17(SRC)
PTPN6
PTPRA
LYN
Y409(CTTN)
Y384(CTTN)
S368(CTTN)
S381(CTTN)
S69(SRC)
S371(GIT1)
Y508(LYN)
S11(LYN)
Y674(CBL)
CBL
GIT1
ARHGEF6
PARVB
T277(PARVB)
INPPL1
Y886(INPPL1)
Y986(INPPL1)
OSTF1
S213(OSTF1)
S822(ASAP2)
ASAP2
S701(ASAP2)
PTK2B
S375(PTK2B)
S184(ABI1)
ABI1
S684(ARHGEF6)
Y110(NCK2)
NCK2
Y342(NCK2)
