# Supplementary material for: Phosphoproteomic Analysis of Platelets Activated by Pro-Thrombotic Oxidized Phospholipids and Thrombin
Source: PLoS One. 2014 Jan 6;9(1):e84488. doi: 10.1371/journal.pone.0084488 (PMC3882224; doi:10.1371/journal.pone.0084488)
Supplement: Materials and Methods S1 — Detailed description of trypsin digestion of the protein lysate, phosphopeptide enrichment, mass spectrometry analysis, chromatography alignment, quantitation, and bioinformatics and enrichment analysis employed to study the phosphoproteome changes induced by the agonists. (PDF) [file pone.0084488.s003.pdf]

MATERIALS AND METHODS S1

1. Phosphoproteomic analysis of human platelets

Phosphoproteome changes induced by the agonists were assessed based on trypsin digestion of the protein lysate, phosphopeptide enrichment, mass spectrometry analysis, chromatography alignment, and quantitation. An additional description of the protocol containing useful tips for each of the steps is found elsewhere [1].

1.1. Platelet treatment and lysis

Human platelets isolated by gel filtration (2.7×10^8 / mL) were incubated in Tyrode's buffer with 50 µM KODA-PC or PLPC (as control) for 30 min at 37 °C. The same protocol was applied when studying platelet activation by thrombin, where gel-filtered platelets were incubated in Tyrode's buffer with 0.05 U/mL thrombin or in buffer alone (Resting) for 3 min at 37 °C. Platelets were then centrifuged at 3,700 g (10 min, 35 °C) and lysed in 8 M urea, 50 mM Tris-HCl (pH 7.4), 1 mM Na_3VO_4, and 1 mM NaF with sonication. Lysate was cleared by centrifugation at 3,500 g for 15 min followed by filtration through 0.45 µm and 0.22 µm filter units. Protein concentration in each sample was determined using Bradford assay (Bio-Rad). The initial amount of platelet-derived protein per sample was 11 mg.

1.2. Alkylation, digestion, and reverse phase extraction

1 M sodium phosphate (pH 7.5) was added to the lysate to a 0.1 M final concentration. Alkylation of cysteine residues was achieved by incubation with 5 mM DTT (Bio-Rad) for 1 h at 37 °C followed by 25 mM iodoacetamide for 45 min in the dark, and 10 mM DTT for 30 min at room temperature. Protein lysate was dialyzed for 4 hours with 2 M urea, 50 mM Tris base, and glacial acetic acid to pH 8.0. Sample was then adjusted to pH 7.5 – 8 with 1 M Tris base and digested with TPCK treated trypsin (Worthington, Lakewood, NJ) at 1:100 trypsin/protein ratio for 2 hours at 37 °C followed by sequencing grade modified

trypsin (Promega, Madison, WI) at 1:100 trypsin/protein ratio with overnight incubation at 37 °C. Digested lysate was filtered by centrifugation with Amicon Ultra-15 Centrifugal Filter Unit (Millipore, Billerica, MA) and acidified with 5 % TFA to pH 3.5-4.5. Sample was then loaded into a C18 SPE tube (Discovery® DSC-18 SPE tube, 500 mg bead weight, Sigma, St. Louis, MO), washed with 2 column volumes of 0.1 % TFA in water, and eluted with 40 % acetonitrile, 0.1 % TFA. Solvents are removed to dryness by overnight lyophilization.

1.3. Immunoprecipitation of tyrosine-phosphorylated peptides and enrichment with iron metal affinity media

Lyophilized peptides are dissolved in 100 mM Tris-HCl (pH 8) and the pH adjusted to 7.4 with 1 M Tris base prior to the addition of 150 μL (pre-washed 50% slurry) anti-phosphotyrosine clone 4G10® agarose conjugate (Millipore) and incubated overnight at 4 °C [2,3]. After immunoprecipitation of tyrosine-phosphorylated peptides we kept the supernatant for the enrichment of serine/threonine peptides (Section 1.4). Immunoprecipitated material is washed with 450 μL 50 mM Tris-HCl (pH 7.4) three times, 450 μL 25 mM NH_4HCO_3 twice, and finally the peptides are eluted with 500 μL 0.1 % TFA for 15 min at 37 °C. Sample is dried by vacuum centrifugation and then dissolved in 500 μL 250 mM acetic acid, 30 % acetonitrile. Phosphorylated peptides are further enriched with 60 μL PHOS-Select™ Iron Affinity Gel (Sigma, St. Louis, MO) for 45 min at room temperature mixing continuously [4]. PHOS-Select beads are washed with 250 μL 250 mM acetic acid, 30 % acetonitrile twice followed by 250 μL water. Peptides are eluted with 250 μL 1.6 % NH_3 in water for 5 min at room temperature. Ammonia is removed by vacuum centrifugation and the sample is cleaned up for mass spectrometry analysis with MonoTip C18, 200 μL volume (GL Sciences, Torrance, CA) following manufacturer's instructions.

1.4. Affinity enrichment of serine/threonine-phosphorylated peptides

For the enrichment of serine/threonine phosphorylated peptides we used the supernatant remaining after immunoprecipitation of phospho-tyrosine peptides (Section 1.3). The supernatant was acidified with 5 % TFA to pH 3.5-4.5. Sample was then loaded into a C18 SPE tube as described above (Section 1.2) and the solvent removed to dryness by overnight lyophilization. Peptides are dissolved in 2 mL buffer 5 mM KH_2PO_4 (pH 2.65), 30% acetonitrile, 5 mM KCl and loaded into PolySULFOETHYL-A SPE cartridges (Poly LC, Columbia, MD) for fractionation of peptides based on their charge at pH 2.65 [5-8]. The eluate is collected right away by adding an additional 2 mL of 5 mM KH_2PO_4 (pH 2.65), 30 % acetonitrile, 5 mM KCl followed by 4 mL 5 mM KH_2PO_4 (pH 2.65), 30 % acetonitrile, 17.5 mM KCl. This eluate will be termed **LWF1**. An additional fraction (designated **F2**) is collected with 4 mL of 5 mM KH_2PO_4 (pH 2.65), 30 % acetonitrile, 70 mM KCl. Acetonitrile from all fractions is removed by vacuum centrifugation prior to loading each fraction into C18 SPE tubes as indicated in section 1.2. After washing the SPE cartridge with 0.1 % TFA, the peptides are eluted with 50% acetonitrile, 0.1% TFA and lactic acid (Fluka, Sigma, St. Louis, MO) added to all fractions to a final concentration 150 mg/mL [9]. Phosphorylated peptides are enriched by adding titanium dioxide [10] (Poly LC, Columbia, MD) to each fraction and mixing continuously for 45 min at room temperature. Titania beads are washed with 45 % acetonitrile, 0.1 % TFA, 150 mg/ml lactic acid three times, and 45 % acetonitrile, 0.1 % TFA twice. Phosphorylated peptides are eluted with 3 % NH_3 in water for 5 min at room temperature. Eluted material is concentrated by vacuum centrifugation until dry and the samples cleaned up for mass spectrometry analysis with MonoTip C18, 200 µL volume (GL Sciences, Torrance, CA) following manufacturer's instructions.

1.5. Mass spectrometry analysis

Phosphorylated peptides are analyzed by LC-MS/MS with an Eksigent autosampler coupled with NanoLC 2D pump (Eksigent, Dublin, CA) and LTQ-Orbitrap (Thermo Fisher Scientific, Waltham, MA). Samples loaded onto an analytical column (10 cm × 75 µm i.d.) packed with 5 µm Integrafit Proteopep2

300 Å C18 (New Objective, Woburn, MA). Peptides are eluted into the mass spectrometer using a HPLC gradient of 5-40 % Buffer B in 45 min followed by a quick gradient of 40-90 % Buffer B in 10 min, where Buffer A contains 0.1% formic acid in water and Buffer B contains 0.1% formic acid in acetonitrile. Mass spectra are collected in positive ion mode using the Orbitrap for parent mass determination and the LTQ for data dependent MS/MS acquisition of the top 5 most abundant peptides. MS/MS fragmentation spectra were searched with the Sequest algorithm within the Proteome Discoverer software framework (Thermo Fisher Scientific, version 1.3) against a human UniProt database (version released January, 2012, downloaded from www.uniprot.org, 73,842 sequences in total). The search parameters included: 2 maximum missed trypsin cleavage sites, 15 ppm precursor mass tolerance, 0.8 Da fragment mass tolerance, carbamidomethyl-Cys as a static modification, and oxidation of Met and phosphorylation of Ser/Thr/Tyr as dynamic modifications. Significance scoring of identified peptides was done with the Percolator node of Proteome Discoverer, which uses a support vector machine model trained on actual and decoy search results [11]. We filtered the dataset for a peptide match FDR < 0.01. Proteins were collapsed to protein groups using the protein grouping algorithm of Proteome Discoverer considering the "*strict maximum parsimony principle*",*"only PSMs [peptide spectrum matches] with confidence at least medium"*, and *"only PSMs with delta Cn better than 0.15"*. The confidence of phosphorylation site localization was assessed with the phosphoRS 2.0 node within the Proteome Discoverer framework [12]. Finally, for more consistent reporting of phosphorylation sites (phosphorylation positions) we remapped each identified phosphopeptide to the largest matching protein of the uniprot database. Additional filtering after Proteome Discoverer included ppm ≤ 3, and PEP ≤ 0.05. Peptide information (phosphoRS site probabilities, DeltaCn, PEP, XCorr, ppm, MH+ (Da), and matched ions) included in Supplemental Table 1 was selected from redundant assignments of a phosphopeptide with the same sequence, charge, and methionine oxidation state based on the lowest PEP value.

1.6. Chromatography profile alignment and peak identification based on alignment

Phosphopeptide peaks sequenced in some samples but not others (common in data-dependent MS2 fragmentation sequencing) were located through the alignment of chromatogram elution profiles with a dynamic time warping algorithm [13]. An explanation of the algorithm performance for phosphopeptide peak identification when the peak is not sequenced by data-dependent MS2 fragmentation can be found in the supporting information of our previous publications [6,14]. Results from the alignment were inspected individually and peptides with poor chromatography were eliminated from further quantitative analysis. Comparison between label-free and SILAC-based quantitation demonstrated a good correlation in the results obtained by both quantitative approaches [14].

1.7. Quantitation of phosphorylation responses in platelets activated by KODA and thrombin

Single biological experiments (KODA-induced *vs.* PLPC-induced and Thrombin-induced *vs.* resting platelets) were analyzed by LC-MS/MS twice (replicate runs) and the average peak area and coefficient of variation were calculated after chromatography alignment. Ratios of phosphopeptide peak areas were calculated by dividing the average peak intensity from KODA-treated platelets by the average peak area from PLPC- treated platelets. A fold change ≥ 1.5 (or ≤ 0.66 for dephosphorylation) between KODA-PC-treated and control PLPC-treated platelets and a coefficient of variation lower than 0.3 between replicate runs were used as cutoff values to enrich for phosphorylation events involving a substantial change in the relative amounts of the phosphopeptide induced by the agonist. In cases when phosphopeptides of the same sequence were detected in multiple ionization charge or methionine oxidation states, we required consistency across all detected peptide states, namely a fold change ≥1.5 (or ≤ 0.66 for dephosphorylation) and a coefficient of variation lower than 0.4 between replicate runs for all detected peptide charge and oxidation states. The same thresholds were applied for Thrombin-treated platelets and resting platelets.

2. Bioinformatic analysis of phosphoproteomic data

2.1. Hierarchical clustering

We obtained a complete list of all the KODA-induced and/or Thrombin-induced phosphorylation events that were detected in both experiments and calculated the log_2 fold for representation purposes (Figure 2A). Data was clustered using an uncentered correlation for genes (in this case protein and phosphorylation site) and single linkage as the clustering method. The Cluster and TreeView programs were used to cluster and visualize the data [15].

2.2. Motif-X

Enriched phosphorylation motifs were extracted from significantly upregulated or downregulated phosphorylation events for sequences (confidence probability assignment > 90%) using the web-based software Motif-X (http://motif-x.med.harvard.edu/motif-x.html) [16]. Sequences were centered for each phosphorylation site for 13 aa width, with a minimum of 2 occurrences and 0.00021 significance (p-value 0.05). Human IPI proteome background was provided by the website. Classification of kinase/phosphatase or binding motifs was done using PhosphoMotif Finder [17].

2.3. Kyoto Encyclopedia of Genes and Genomes (KEGG) pathway and Gene Ontology (GO)-term annotations

Pathway enrichment analysis for Kyoto Encyclopedia of Genes and Genomes (KEGG) pathway and GO-term annotations were done using DAVID Bioinformatic Resources [18]. For this analysis, the term "gene list" refers to the list of proteins with significant differences in phosphorylation between PLPC and KODA-PC or Resting and Thrombin samples in at least one phosphorylation site as indicated in Supplemental Table 1; the term "background list" refers to the complete list of proteins identified by mass

spectrometry (PLPC- and KODA-PC-treated or resting and thrombin-treated samples) as listed in Supplemental Table 1.

2.4. Kinase enrichment analysis

We used KEA [19] (Kinase enrichment analysis) for the prediction of kinases responsible for phosphorylation of proteins induced by KODA-PC or thrombin. Entrez gene symbols from KODA- and Thrombin-induced (de)phosphorylated proteins (Supplemental Table 1) are used as input for the web-based kinase enrichment analysis.

2.5. Construction of the phosphoproteome integrin adhesome

The phosphoproteome network for integrin activation was constructed by matching phosphoproteomic results from Thrombin-activated platelets with an integrin adhesome database [20] (downloaded from http://www.adhesome.org/interactions/index.htm). Additional protein-protein interactions [21,22] were added following the original database annotations. Source and target connections were extracted from the integrin adhesome database. Sites of phosphorylation and trend (i.e. phosphorylation, de-phosphorylation, or no change) were obtained from Supplemental Table 1. The compiled data was plotted using Cytoscape [23] for network visualization. Distribution of nodes was manually arranged for better depiction of the data.

REFERENCES

1. Zimman A, Berliner JA, Graeber TG (2013) Phosphoproteomic analysis of aortic endothelial cells activated by oxidized phospholipids. Methods Mol Biol 1000: 53-69.
2. Rush J, Moritz A, Lee KA, Guo A, Goss VL, et al. (2005) Immunoaffinity profiling of tyrosine phosphorylation in cancer cells. Nat Biotechnol 23: 94-101.
3. Skaggs BJ, Gorre ME, Ryvkin A, Burgess MR, Xie Y, et al. (2006) Phosphorylation of the ATP-binding loop directs oncogenicity of drug-resistant BCR-ABL mutants. Proc Natl Acad Sci U S A 103: 19466-19471.
4. Stensballe A, Andersen S, Jensen ON (2001) Characterization of phosphoproteins from electrophoretic gels by nanoscale Fe(III) affinity chromatography with off-line mass spectrometry analysis. Proteomics 1: 207-222.
5. Villen J, Beausoleil SA, Gerber SA, Gygi SP (2007) Large-scale phosphorylation analysis of mouse liver. Proc Natl Acad Sci U S A 104: 1488-1493.
6. Zimman A, Chen SS, Komisopoulou E, Titz B, Martinez-Pinna R, et al. (2010) Activation of aortic endothelial cells by oxidized phospholipids: a phosphoproteomic analysis. J Proteome Res 9: 2812-2824.
7. Dephoure N, Gygi SP (2011) A solid phase extraction-based platform for rapid phosphoproteomic analysis. Methods 54: 379-386.
8. Olsen JV, Blagoev B, Gnad F, Macek B, Kumar C, et al. (2006) Global, in vivo, and site-specific phosphorylation dynamics in signaling networks. Cell 127: 635-648.
9. Sugiyama N, Masuda T, Shinoda K, Nakamura A, Tomita M, et al. (2007) Phosphopeptide enrichment by aliphatic hydroxy acid-modified metal oxide chromatography for nano-LC-MS/MS in proteomics applications. Mol Cell Proteomics 6: 1103-1109.
10. Larsen MR, Thingholm TE, Jensen ON, Roepstorff P, Jorgensen TJ (2005) Highly selective enrichment of phosphorylated peptides from peptide mixtures using titanium dioxide microcolumns. Mol Cell Proteomics 4: 873-886.
11. Kall L, Canterbury JD, Weston J, Noble WS, MacCoss MJ (2007) Semi-supervised learning for peptide identification from shotgun proteomics datasets. Nat Methods 4: 923-925.
12. Taus T, Kocher T, Pichler P, Paschke C, Schmidt A, et al. (2011) Universal and confident phosphorylation site localization using phosphoRS. J Proteome Res 10: 5354-5362.
13. Prakash A, Mallick P, Whiteaker J, Zhang H, Paulovich A, et al. (2006) Signal maps for mass spectrometry-based comparative proteomics. Mol Cell Proteomics 5: 423-432.
14. Rubbi L, Titz B, Brown L, Galvan E, Komisopoulou E, et al. (2011) Global phosphoproteomics reveals crosstalk between Bcr-Abl and negative feedback mechanisms controlling Src signaling. Sci Signal 4: ra18.
15. Eisen MB, Spellman PT, Brown PO, Botstein D (1998) Cluster analysis and display of genome-wide expression patterns. Proc Natl Acad Sci U S A 95: 14863-14868.
16. Schwartz D, Gygi SP (2005) An iterative statistical approach to the identification of protein phosphorylation motifs from large-scale data sets. Nat Biotechnol 23: 1391-1398.
17. Amanchy R, Periaswamy B, Mathivanan S, Reddy R, Tattikota SG, et al. (2007) A curated compendium of phosphorylation motifs. Nat Biotechnol 25: 285-286.
18. Dennis G, Jr., Sherman BT, Hosack DA, Yang J, Gao W, et al. (2003) DAVID: Database for Annotation, Visualization, and Integrated Discovery. Genome Biol 4: P3.
19. Lachmann A, Ma'ayan A (2009) KEA: kinase enrichment analysis. Bioinformatics 25: 684-686.
20. Zaidel-Bar R, Itzkovitz S, Ma'ayan A, Iyengar R, Geiger B (2007) Functional atlas of the integrin adhesome. Nat Cell Biol 9: 858-867.
21. Malinin NL, Plow EF, Byzova TV (2010) Kindlins in FERM adhesion. Blood 115: 4011-4017.
22. Harburger DS, Calderwood DA (2009) Integrin signalling at a glance. J Cell Sci 122: 159-163.
23. Smoot ME, Ono K, Ruscheinski J, Wang PL, Ideker T (2011) Cytoscape 2.8: new features for data integration and network visualization. Bioinformatics 27: 431-432.
